# Sustainability of affordable housing programs in Kenya: A case of Meru and Embu Counties

**DOI:** 10.1371/journal.pone.0343915

**Published:** 2026-07-16

**Authors:** Karambu Kiende Gatimbu, Paul Njiru Nthakanio

**Affiliations:** 1 Department of Business Studies, University of Embu, Embu, Kenya; 2 Department of Water, Agriculture and Resource Management, University of Embu, Embu, Kenya; North Carolina State University, UNITED STATES OF AMERICA

## Abstract

Amid rising urbanization and population growth, Kenya faces a critical housing shortage that disproportionately affects low- and middle-income populations. In response, the government launched the Affordable Housing Program (AHP) under the Big Four Agenda to address this housing gap. This study evaluates the sustainability of AHP projects in Meru and Embu counties, focusing on economic, social, and environmental dimensions. Using a descriptive research design, data were collected from 215 purposively selected participants comprising members of the public, academics, and contractors. Quantitative data were analyzed using Stata 18, including descriptive statistics, factor analysis, and Generalised Linear Models (GLMs). The results indicate that while the AHP has made significant progress in improving housing affordability and social safety, challenges remain, particularly in environmental sustainability, stakeholder engagement, and the use of local materials. Safety, security, and affordability ranked highest across respondents, whereas features such as lift access and energy-efficient systems were ranked lower. The findings also highlight demographic differences in sustainability preferences: older respondents emphasized safety and infrastructure reliability, whereas postgraduate respondents prioritized environmental sustainability. The study recommends stronger stakeholder involvement, localization of policy frameworks, and integration of green building standards. These insights provide valuable guidance for policymakers and practitioners seeking to scale sustainable housing solutions in Kenya and similar developing contexts.

## Introduction

Access to affordable, adequate, and sustainable housing has become one of the most pressing development challenges in the Global South, especially in rapidly urbanizing countries such as Kenya. According to [1], urban populations in Africa are projected to triple by 2050, intensifying the demand for affordable housing. Kenya’s housing deficit currently stands at over two million units, growing annually by approximately 200,000 units [2,3]. The pressure to address this shortage led to the launch of Kenya’s Affordable Housing Program (AHP), one of the pillars of the Big Four Agenda initiated in 2017. The program forms part of a medium-term development strategy aligned with Kenya Vision 2030 and was established by the Government of Kenya to stimulate economic development and improve access to decent and affordable housing.

Despite its ambitious goals, the Affordable Housing Program (AHP) faces several interrelated challenges that may undermine its effectiveness and long-term success. Affordability remains a major concern, with housing costs often exceeding the financial capacity of many low- and middle-income households, who constitute the primary target beneficiaries [4,5]. In addition, inadequate financing mechanisms, limited access to affordable mortgage products, land acquisition constraints, bureaucratic delays, and inconsistent provision of essential services such as water, sanitation, and electricity continue to hinder successful implementation [6,7]. Moreover, sustainability concerns, including poor environmental integration and inadequate community participation, have raised questions about the program’s long-term impact [8].

In this study, sustainability is conceptualized as a multidimensional construct encompassing economic viability, social inclusivity, and environmental stewardship, consistent with the widely recognized three-pillar framework of sustainable development advanced by the United Nations and the World Commission on Environment and Development [9,10]. Accordingly, sustainability extends beyond the physical durability of housing structures to include long-term affordability, equitable access to housing and basic services, community cohesion, and the adoption of environmentally responsible construction and management practices.

Kenya’s housing policies have historically been centralized and infrastructure-focused, leaving little room for localized solutions and community-driven initiatives [11]. This top-down approach often creates a mismatch between policy design and local realities, thereby undermining program effectiveness [1,12]. Furthermore, access to basic services such as clean water, sanitation, and electricity remains inconsistent in many public housing projects, particularly outside major urban centres [7,12].

This study situates itself within this context by examining the sustainability of the AHP through a three-pronged lens: economic, social, and environmental sustainability. Economic sustainability examines whether the housing units are affordable to the target groups and whether construction costs are aligned with local income levels. Social sustainability assesses whether the housing projects promote inclusive communities with access to essential services and a sense of safety and security. Environmental sustainability investigates whether these housing units employ energy-efficient technologies, environmentally friendly materials, and effective waste management systems [1,13].

Meru and Embu counties serve as ideal case study areas due to their varying socioeconomic profiles, semi-urban characteristics, and active AHP implementation. Meru has a more agriculturally driven economy, while Embu has experienced relatively faster urban expansion. These differences provide an opportunity to explore regional variations in the perception and implementation of sustainable housing [7,12].

Furthermore, this study incorporates demographic factors such as age, gender, education level, and professional background to understand how different stakeholders perceive sustainability. Prior studies have shown that demographic factors significantly influence sustainability preferences [14,15]. More recent studies further highlight the role of knowledge, socio-economic status, and behavioural factors [16,17]. For example, older individuals may prioritize physical security, while younger or more educated respondents may emphasize environmental features such as renewable energy and green spaces [15,18].

By employing a mixed-methods approach that combines structured questionnaires, literature review, and policy analysis, this research provides a comprehensive evaluation of the AHP’s sustainability. In doing so, it contributes to the academic discourse on sustainable housing and informs policy formulation and program design for future housing initiatives in Kenya and other developing countries. The remainder of the paper is structured as follows: the *Literature Review* section examines existing studies on housing sustainability and policy frameworks; the *Materials and Methods* section describes the study design, data collection, and analytical techniques; the *Results and Discussion* section presents and interprets the findings; and the *Conclusion and Recommendations* section provides the final synthesis and policy implications.

## Literature review

### Theoretical framework

Sustainable housing is grounded in the broader concept of sustainable development, commonly defined as development that meets present needs without compromising the ability of future generations to meet their own needs [19]. This concept has since evolved into a guiding principle for planning and policy across multiple sectors, including housing.

The theoretical foundation of this study is based on the triadic model of sustainability, which comprises economic, social, and environmental dimensions. This framework provides a holistic lens for evaluating long-term housing viability and has been widely adopted in sustainability research and policy [10]. The economic dimension focuses on affordability and financial accessibility; the social dimension emphasizes inclusivity, safety, and community well-being; and the environmental dimension addresses resource efficiency, resilience, and ecological impact.

This tripartite framework has become foundational in housing sustainability assessments globally. For instance, according to [20], sustainability in housing should integrate affordability (economic), inclusivity and safety (social), and environmental resilience. Similarly, [21] emphasizes that these pillars are interdependent and interact dynamically, requiring integrated, context-specific strategies for effective implementation. Recent studies further highlight that sustainable housing must respond to climate change, urbanization pressures, and socio-economic inequalities, particularly in developing countries [17,22].

### Conceptual framework

This study conceptualizes sustainable housing as a multidimensional construct comprising economic, social, and environmental sustainability dimensions. These three pillars constitute the core explanatory variables that influence overall housing sustainability outcomes, consistent with established sustainability frameworks [10].

Within this framework, sustainability outcomes are assessed through indicators such as affordability, access to basic services, housing quality, environmental performance, and user satisfaction. The economic dimension captures housing affordability, cost efficiency, and financial accessibility; the social dimension reflects inclusivity, safety, and access to infrastructure; while the environmental dimension focuses on resource efficiency, energy use, and ecological impact [1,21].

Demographic characteristics, specifically age, gender, education level, and professional role, are treated as mediating variables that influence how individuals perceive and prioritize these sustainability dimensions. Empirical evidence shows that demographic factors shape housing preferences, environmental awareness, and affordability thresholds, thereby influencing sustainability evaluations [16,18].

The conceptual model, therefore, assumes that sustainability perceptions and housing outcomes are not uniform but are moderated by socio-demographic characteristics. This interaction provides a more nuanced understanding of housing sustainability by linking structural housing attributes with user-specific preferences and socio-economic realities.

### Research gaps and emerging needs

While the academic and policy literature on affordable housing in Kenya has grown, several critical gaps remain. Little empirical research has been conducted on AHP in Meru and Embu counties [7,12]. There is a significant gap in studies evaluating the ecological sustainability of housing projects, particularly concerning green energy use and sustainable construction materials [1,13]. Limited research explores the influence of participatory planning and community engagement on project sustainability, despite its importance in enhancing ownership and long-term project success [1,23]. Most studies focus solely on economic sustainability, neglecting the interconnectedness of social and environmental factors that define true housing sustainability [1,13].

This study aims to fill these gaps by conducting a localized, multidimensional assessment that integrates demographic analysis and statistical modelling. It contributes to the growing call for data-driven, regionally informed, and socially inclusive housing policy frameworks in Kenya [24].

Despite the growing body of research, several gaps persist. First, most studies focus on Nairobi and Mombasa, neglecting semi-urban counties like Meru and Embu [7,12]. Second, there is limited exploration of how demographic variables influence sustainability perceptions, particularly across different stakeholder groups such as households, professionals, and local authorities [25]. Third, existing evaluations rarely triangulate economic, social, and environmental dimensions, often treating them in isolation rather than as an integrated sustainability framework [13]. Moreover, while sustainability policies exist on paper, few studies have measured their implementation effectiveness at the county level [12]. There is also minimal attention paid to the role of professional stakeholders such as contractors and academics in shaping housing outcomes and policy translation processes [23].

This study addresses these gaps by combining empirical data with stakeholder perspectives across two counties. It evaluates sustainability holistically and provides evidence-based recommendations for regional policy design.

## Materials and methods

This section outlines the research design, study area, population, sampling procedures, data collection instruments, and analytical methods used in the study. The methodology was structured to ensure rigour, reliability, and validity in assessing the sustainability of the Affordable Housing Program (AHP) in Meru and Embu counties.

### Research design

The study adopted a descriptive cross-sectional survey design. Descriptive research is widely applied when the objective is to systematically describe characteristics of a population or phenomenon at a specific point in time [19]. This design was appropriate as it enabled the collection of data from a relatively large sample within a limited timeframe while examining relationships between demographic characteristics and perceptions of economic, social, and environmental sustainability within the AHP.

A cross-sectional approach was further justified because the study focused on capturing current perceptions, experiences, and evaluations without manipulating any variables [26]. Such designs are commonly used in housing and urban studies to obtain real-time stakeholder perspectives and contextual insights [27].

### Study area and justification

The study was conducted in Meru and Embu counties, which are among the regions actively implementing the Affordable Housing Program. The two counties were selected due to their differing socio-economic, spatial, and infrastructural characteristics, which provide a suitable basis for comparative analysis.

Meru County exhibits a mixed urban–rural structure with moderate urban growth and ongoing housing developments. In contrast, Embu County is more semi-urban and has experienced relatively rapid urban expansion driven by population growth and infrastructure development [28]. These differences reflect distinct stages of urban development, governance structures, and service delivery capacities, making the two counties suitable for comparative sustainability analysis.

### Target population and sampling technique

The target population comprised stakeholders directly or indirectly involved in the Affordable Housing Program (AHP), including public sector officials such as housing officers and urban planners, academic experts in housing and urban studies, private sector actors including architects, engineers, and contractors, as well as community representatives and prospective housing beneficiaries within Embu and Meru counties. The inclusion of respondents from different stakeholder groups was intended to capture diverse perspectives on the social, economic, and environmental dimensions of housing sustainability.

Purposive sampling was employed to select respondents with relevant knowledge, experience, or engagement in housing-related activities. This non-probability sampling technique was considered appropriate because the study sought information-rich participants capable of providing informed assessments of sustainability indicators within the AHP framework [29]. The approach also enabled the inclusion of respondents from policy, technical, and community contexts, thereby broadening the perspectives represented in the study.

A total of 215 respondents participated in the survey, comprising 132 respondents from Embu County and 83 respondents from Meru County. The variation in sample distribution reflected differences in housing activity levels, urban development patterns, and population concentration between the two counties. The final sample included respondents from public institutions (46.3%), academia (36.4%), and the construction sector (17.3%), as well as community-level perspectives from individuals familiar with local housing conditions and residential challenges. This composition strengthened the study by integrating policy, technical, and user-oriented viewpoints in the assessment of housing sustainability.

### Instrumentation and data collection tools

Data were collected using a semi-structured questionnaire developed from established housing sustainability instruments [30]. The questionnaire consisted of five sections: demographic information; economic sustainability indicators (including affordability, household income, and construction costs); social sustainability indicators (including safety, tenure security, and access to services); environmental sustainability indicators (including use of green materials, energy efficiency, and waste management); and overall perceptions of program performance and satisfaction.

The instrument included Likert-scale items (1–5) and open-ended questions to capture qualitative insights. Respondents were also asked to rank their priorities across the three sustainability dimensions to support comparative analysis.

To enhance validity and clarity, the instrument was pilot-tested with 20 respondents in Tharaka Nithi County. Based on feedback, ambiguous items were refined, and the questionnaire layout was improved for clarity and usability.

### Secondary data sources

Secondary data were obtained through document review and desk research. Sources included national housing policy documents, Kenya Vision 2030, the Big Four Agenda reports, County Integrated Development Plans, peer-reviewed journal articles, books, and credible institutional reports published between 2019 and 2024. Additional data were drawn from UN-Habitat and World Bank housing program assessments. These sources provided contextual grounding, supported questionnaire development, and informed comparative interpretation of findings.

### Data collection procedure

Data collection was conducted over six weeks (February–March 2021). Questionnaires were administered face-to-face and online. Face-to-face administration was primarily used for public officials and community representatives, while online questionnaires (Google Forms) were distributed to academics and construction professionals due to logistical constraints.

Research assistants were trained to ensure consistency in data collection procedures and adherence to ethical standards. Regular supervision and follow-up checks were conducted to ensure completeness and accuracy of responses. The study involved non-sensitive survey data on housing perceptions. It did not include clinical, biomedical, or other high-risk human-subject research requiring invasive procedures or the collection of personal health information. Participation was entirely voluntary, and all respondents provided informed consent. Confidentiality and anonymity of respondents were maintained throughout the study, and no personally identifiable information was collected or disclosed.

### Reliability and validity of the instrument

The questionnaire’s reliability was assessed using Cronbach’s alpha, in accordance with established methodological standards [31,32]. The reliability coefficients for the constructs were as follows: economic sustainability (α = 0.86), social sustainability (α = 0.81), and environmental sustainability (α = 0.79). All values exceeded the recommended threshold of 0.70, indicating satisfactory internal consistency [33].

Content validity was established through expert review by three housing specialists, a sociologist, and an environmental planner. According to [34,35], construct validity is further supported by an exploratory factor analysis (EFA), which, in this study, confirmed that the sustainability dimensions loaded distinctly with minimal cross-loadings, thereby validating the measurement structure.

### Data analysis techniques

Quantitative data were analyzed using Stata version 18. Descriptive statistics, including means, frequencies, and standard deviations, were computed to summarise respondent characteristics and responses across the economic, social, and environmental sustainability dimensions.

An Exploratory Factor Analysis (EFA) was conducted to identify the latent constructs underlying participants’ perceptions of sustainability indicators. The EFA model is expressed as:


$$\begin{array}{c} X_{i}=\lambda_{i\text{1}}F_{\text{1}}+\lambda_{i\text{2}}F_{\text{2}}+\cdots +\lambda_{im}F_{m}+\epsilon_{i} \end{array}$$ (1)

where $X_{i}$ represents observed variables, $F_{m}$ are latent factors, $\lambda_{im}$ are factor loadings, and $\epsilon_{i}$is the error term. The suitability of the data for factor analysis was assessed using the Kaiser–Meyer–Olkin (KMO) measure of sampling adequacy and Bartlett’s Test of Sphericity. Factors were extracted using principal axis factoring and rotated using varimax rotation to enhance interpretability. EFA was appropriate for this study as it enables the identification of underlying structures among multiple correlated variables, making it widely used in social science research [36,37].

Principal Component Analysis (PCA) was subsequently applied to reduce the identified variables into a smaller set of uncorrelated components within each sustainability dimension. Each principal component is a linear combination of the original variables:


$$\begin{array}{c} PC_{j}=a_{j\text{1}}X_{\text{1}}+a_{j\text{2}}X_{\text{2}}+\cdots +a_{jp}X_{p} \end{array}$$ (2)

where $a_{jp}$ are component loadings, components with eigenvalues greater than 1 were retained, following Kaiser’s criterion. PCA was selected to simplify the dataset and support the construction of composite indices, which is particularly useful in studies involving multidimensional constructs such as sustainability [38].

Generalized Linear Models (GLMs) were employed to examine the influence of demographic variables (age, gender, profession, and education level) on sustainability ratings. The general GLM specification is:


$$\begin{array}{c} g(E[Y])=\beta_{\text{0}}+\beta_{\text{1}}X_{\text{1}}+\beta_{\text{2}}X_{\text{2}}+\cdots +\beta_{k}X_{k} \end{array}$$ (3)

where g(·) is the link function, Y is the response variable, and $X_{k}$ are predictor variables. A Gaussian family with an identity link function was specified for continuous outcomes. GLMs were chosen for their flexibility in handling non-normal response distributions and for their suitability for modelling Likert-scale data as approximately continuous variables [39,40].

Independent samples t-tests and one-way Analysis of Variance (ANOVA) were used to assess statistically significant differences in sustainability perceptions across counties and stakeholder groups. These techniques are appropriate because the study involves comparing mean sustainability perception scores across independent categorical groups, with the dependent variables (economic, social, and environmental sustainability indices) measured on a continuous Likert-scale composite index.

The independent samples t-test is suitable for comparing the means of two independent groups, such as Meru and Embu counties, to determine whether observed differences in sustainability perceptions are statistically significant rather than due to random variation. For comparisons involving more than two groups (e.g., age categories, education levels, and professional roles), one-way ANOVA was employed to test whether at least one group mean differs significantly from the others. ANOVA is preferred in such cases because it controls for Type I error inflation that would arise from conducting multiple pairwise t-tests.

Post hoc tests (Tukey’s HSD) were conducted where applicable to identify specific group differences after a significant ANOVA result. These parametric tests are appropriate under the assumption that the data are approximately normally distributed, observations are independent, and variances are homogeneous across groups, conditions that are reasonably satisfied for aggregated Likert-scale indices in large samples [41].

Qualitative data from open-ended responses were analyzed using thematic analysis. Responses were coded inductively and deductively using NVivo version 12. Initial codes were generated, grouped into categories, and refined into overarching themes aligned with the sustainability framework. Thematic analysis was selected for its flexibility in identifying patterns across qualitative datasets and its suitability for mixed-methods research [42].

### Results

This section presents findings derived from questionnaire data collected in Meru and Embu counties. Results are organized into three sustainability dimensions: social, economic, and environmental. In addition, multivariate analyses using Principal Component Analysis (PCA) and Generalised Linear Models (GLMs) are presented to examine latent structures in sustainability indicators and the influence of demographic variables on sustainability perceptions.

### Overview of respondents

A total of 215 respondents participated in the study, of which 62.8% were male, and 37.2% were female, as shown in Table 1. In terms of educational attainment, 37.7% held a bachelor’s degree, while 21.9% had postgraduate qualifications (Master’s, postgraduate diploma, or PhD). Regarding professional background, 46.3% were from the public sector, 36.4% from academia, and 17.3% from the construction sector. The sample included respondents with varying levels of experience in housing-related activities.

**Table 1:** Socio-economic characteristics of respondents.

Parameters		Embu	Meru	Pooled
Sample size		132 (100)	83 (100)	215 (100)
Designation	Academia	49 (37.4)	29 (34.9)	78 (36.4)
	Contractor	15 (11.5)	22 (26.5)	37 (17.3)
	Public	67 (51.1)	32 (38.6)	99 (46.3)
Gender	Male	83 (62.9)	52 (62.7)	135 (62.8)
	Female	49 (37.1)	31 (37.3)	80 (37.2)
Age	Less than 30	36 (27.3)	10 (12)	46 (21.4)
	31-40	42 (31.8)	23 (27.7)	65 (30.2)
	41-50	33 (25)	29 (34.9)	62 (28.8)
	51-60	19 (14.4)	16 (19.3)	35 (16.3)
	Above 60	2 (1.5)	5 (6)	7 (3.3)
Education	Secondary	4 (3)	7 (8.4)	11 (5.1)
	Certificate	17 (12.9)	7 (8.4)	24 (11.2)
	Diploma	32 (24.2)	20 (24.1)	52 (24.2)
	Bachelors	47 (35.6)	34 (41)	81 (37.7)
	Masters	19 (14.4)	11 (13.3)	30 (14)
	Postgraduate	7 (5.3)	3 (3.6)	10 (4.7)
	PhD	6 (4.5)	1 (1.2)	7 (3.3)
Years Worked	< 10 years	65 (49.2)	31 (37.3)	96 (44.7)
	10-15 years	28 (21.2)	30 (36.1)	58 (27)
	16-20 years	11 (8.3)	11 (13.3)	22 (10.2)
	21-25 years	15 (11.4)	8 (9.6)	23 (10.7)
	26-30 years	9 (6.8)	2 (2.4)	11 (5.1)
	> 30 years	4 (3)	1 (1.2)	5 (2.3)

### Social sustainability indicators

The analysis of social sustainability indicators (Table 2) shows that respondents assigned generally high importance to all variables, with mean scores ranging from moderate to very high across both Embu and Meru counties. Overall, safety and security emerged as the most important social criterion (pooled mean = 4.79), followed closely by access to electricity and health facilities (pooled mean = 4.74 for both). These findings indicate that basic service provision and safety considerations remain the primary determinants of perceived housing quality within the Affordable Housing Program (AHP). At the same time, comparatively lower importance was attached to infrastructure and amenity-based features such as lifts (2.95), daycare centres (3.11), and parking space (3.16).

**Table 2:** Social Sustainability Indicators (Mean Scores by County).

Region	Embu mean	Meru mean	Pooled mean	p-value	Embu rank	Meru rank	Pooled rank
Safety/Security	4.72	4.89	4.79	0.022*	1	1	1
Health facilities	4.71	4.8	4.74	0.359	2	7	2
Electricity	4.67	4.86	4.74	0.031*	3	2	2
clean/attractive	4.62	4.82	4.7	0.025*	5	4	4
Pipe borne water	4.64	4.78	4.7	0.098	4	8	4
Location	4.39	4.81	4.55	0.001***	9	5	6
Effective property mngnt	4.36	4.76	4.52	0.001***	10	9	7
Type of building	4.58	4.36	4.49	0.042*	6	15	8
Tenure security	4.58	4.3	4.47	0.022*	6	16	9
Educational centre	4.25	4.83	4.47	<0.001***	14	3	9
Unit size	4.29	4.52	4.38	0.041*	11	13	11
Housing quality	4.55	4.06	4.36	<0.001***	8	19	12
Major/minor access road	4	4.71	4.27	<0.001***	17	10	13
Public transportation	3.91	4.81	4.26	<0.001***	18	5	14
Shopping mall/mkt	3.89	4.59	4.16	<0.001***	19	12	15
Religious places	3.85	4.52	4.11	<0.001***	20	13	16
Privacy	4.27	3.84	4.11	0.002***	12	20	16
Design type	4.15	3.75	4	0.020*	15	21	18
Population density	3.56	4.69	4	<0.001***	29	11	18
Community cohesion	3.7	4.28	3.93	<0.001***	24	17	20
No.bedrooms	4.15	3.4	3.86	<0.001***	15	22	21
Recreational facilities	3.65	4.13	3.84	0.008***	25	18	22
Household size	4.26	3.13	3.82	<0.001***	13	26	23
No.bathrooms	3.78	3.23	3.57	0.001***	21	25	24
Neighbourhood considerations	3.64	3.29	3.5	0.043*	26	23	25
Minimized social segregation	3.62	3.28	3.49	0.080	28	24	26
Neighbourhood reputation	3.64	2.87	3.34	<0.001***	26	27	27
Parking space	3.71	2.28	3.16	<0.001***	23	29	28
Daycare centre	3.75	2.1	3.11	<0.001***	22	30	29
Lift	3.34	2.33	2.95	<0.001***	30	28	30

*p < 0.05, ***p < 0.001.

Marked spatial variations were observed between the two counties. As shown in Table 2, Meru respondents consistently assigned higher importance to accessibility-related indicators such as public transportation (4.81 vs 3.91), access roads (4.71 vs 4.00), and proximity to educational facilities (4.83 vs 4.25), with several of these differences being statistically significant (p < 0.001). In contrast, Embu respondents placed relatively greater emphasis on housing unit and neighbourhood quality attributes, including housing quality (4.55 vs 4.06), tenure security (4.58 vs 4.30), and privacy (4.27 vs 3.84). These patterns suggest that perceptions of social sustainability are strongly influenced by the level of urban development and the availability of infrastructure within each county.

Demographic differences further highlight heterogeneity in perceptions of social sustainability. Older respondents (ages 50+) consistently prioritized safety, infrastructure reliability, and service stability, reflecting concerns about long-term residential security. In contrast, younger respondents (below 35 years) placed relatively greater emphasis on digital readiness, particularly access to Wi-Fi and technology-enabled services. However, these features remain inconsistently integrated within AHP housing units. Overall, the findings presented in Table 2 demonstrate that social sustainability priorities are context-specific and shaped by both spatial development conditions and demographic characteristics, underscoring the need for differentiated housing design and planning approaches.

### Economic sustainability indicators

The results presented in Table 3 indicate that affordability-related considerations primarily influence economic sustainability within the Affordable Housing Program (AHP). Housing affordability recorded the highest pooled mean score (4.80), followed by rental cost relative to income (4.73) and house price relative to income (4.71). These findings suggest that affordability pressures remain central in respondents’ evaluations of housing sustainability. The cost of construction also ranked highly (pooled mean = 4.48), highlighting concerns about rising building and material costs. In contrast, satisfaction of demand (4.25) and mortgage and interest rates (4.20) received comparatively lower ratings, reflecting continued concerns regarding housing finance accessibility and market responsiveness.

**Table 3:** Economic Sustainability Indicators (Mean Scores by County).

Region	Embu mean	Meru mean	Pooled mean	p-value	Embu rank	Meru rank	Pooled rank
House price in relation to income	4.7	4.72	4.71	0.7810	2	6	3
Mortgages & interest rates	4.52	3.7	4.2	0.0000	6	12	12
Rental cost in relation to income	4.69	4.81	4.73	0.1640	3	5	2
Energy bill	4.42	4.06	4.28	0.0070	8	11	10
Transportation cost	4.23	4.66	4.4	0.0010	10	9	7
Employment opportunities	4.09	4.72	4.33	0.0000	11	6	8
Taxation & subsidy	4.43	4.11	4.31	0.0130	7	10	9
Household income	4.54	4.84	4.65	0.0010	5	3	4
Cost of construction	4.24	4.86	4.48	0.0000	9	2	6
Housing affordability	4.75	4.88	4.8	0.0820	1	1	1
Value stability	4.57	4.72	4.63	0.1530	4	6	5
Satisfaction of demand	3.88	4.84	4.25	0.0000	12	3	11

As shown in Table 3, statistically significant differences were observed between Embu and Meru counties for several economic indicators, including cost of construction (Embu = 4.24; Meru = 4.86; p < 0.001), employment opportunities (p < 0.001), transportation cost (p = 0.001), and satisfaction of demand (p < 0.001). Respondents in Meru generally expressed stronger concern regarding supply-side constraints and broader affordability pressures than respondents in Embu. However, housing affordability itself did not differ significantly between the two counties (p = 0.082), suggesting that affordability challenges are broadly shared across the study areas. Overall, the findings in Table 3 demonstrate that economic sustainability in the AHP is constrained not only by housing prices but also by financing accessibility, construction costs, and broader macroeconomic conditions**.**

### Environmental sustainability indicators

The environmental sustainability results presented in Table 4 indicate that respondents placed high importance on environmental quality and resource management indicators within the Affordable Housing Program (AHP). Waste management emerged as the highest-rated environmental indicator, with a pooled mean score of 4.77, followed closely by materials with low environmental impact (4.71), air quality (4.70), use of appropriate materials (4.68), and water pollution control (4.67). These findings suggest that respondents are increasingly aware of the importance of environmentally responsible housing practices and their implications for health, livability, and long-term sustainability. In contrast, indicators associated with advanced sustainability practices, such as in situ energy production (3.27), use of recycled materials (3.26), cement substitutes (3.07), and thermo comfort (2.95), received comparatively lower ratings, indicating limited emphasis or lower implementation of green building technologies within existing housing projects.

**Table 4:** Environmental Sustainability Indicators (Mean Scores by County).

Region	Embu mean	Meru mean	Pooled	p-value	Embu rank	Meru rank	Pooled rank
Air quality	4.64	4.81	4.7	0.082	2	9	3
Waste management	4.71	4.87	4.77	0.052	1	2	1
use of appropriate materials	4.58	4.84	4.68	0.001	4	6	4
Thermo comfort	3.9	1.45	2.95	0.000	17	24	23
Energy efficiency	4.36	3.94	4.2	0.001	12	13	12
Noise pollution	4.39	4.87	4.58	0.000	11	2	8
Water pollution	4.52	4.92	4.67	0.000	9	1	5
Lighting quality	4.55	3.2	4.03	0.000	5	18	14
Natural ventilation	4.54	3.67	4.2	0.000	6	15	12
Toxicity of finishing	4.2	4.75	4.41	0.000	13	12	10
Land use & biodiversity	3.87	4.76	4.21	0.000	18	11	11
Urban density	3.51	4.82	4.01	0.000	22	8	16
Water permeability	4.2	4.78	4.42	0.000	13	10	9
Pre-developed land	3.15	2.63	2.95	0.005	24	20	23
Insitu energy production	3.49	2.9	3.27	0.001	23	19	19
Materials & products reused	3.61	2.58	3.21	0.000	20	21	21
Use of materials with recycled content	3.8	2.41	3.26	0.000	19	22	20
Certified organic materials	4.14	3.71	3.97	0.003	15	14	17
Cement substitute	3.52	2.36	3.07	0.000	21	23	22
Waste management during operation	4.53	4.83	4.65	0.001	8	7	7
Fresh water consumption	4.54	4.86	4.66	0.005	6	5	6
Reuse of grey & rain water	3.99	3.22	3.69	0.000	16	17	18
Materials with low environmental impact	4.61	4.87	4.71	0.003	3	2	2
Regional/local materials	4.4	3.42	4.02	0.000	10	16	15

As shown in Table 4, statistically significant differences were observed between Embu and Meru counties across several environmental indicators (p < 0.05). Meru respondents assigned significantly higher importance to indicators related to pollution control, land use, biodiversity, urban density, and water management. In contrast, Embu respondents placed relatively greater emphasis on lighting quality, natural ventilation, and the use of regional or local materials. Particularly large disparities were observed for thermo comfort (Embu = 3.90; Meru = 1.45; p < 0.001), lighting quality (Embu = 4.55; Meru = 3.20; p < 0.001), and regional or local materials (Embu = 4.40; Meru = 3.42; p < 0.001). These variations suggest differences in environmental priorities, housing conditions, and infrastructure development between the two counties. Furthermore, the relatively low scores for recycled materials and renewable energy indicators suggest limited adoption of circular construction practices and clean energy technologies within the AHP.

Demographic characteristics also significantly influenced perceptions of environmental sustainability. Postgraduate respondents consistently assigned greater importance to environmentally sustainable housing features than respondents with lower levels of education. Similarly, female respondents demonstrated stronger prioritization of environmental quality indicators, particularly waste management, water conservation, and material sustainability. Subsequent GLM analysis confirmed education level as a statistically significant predictor of environmental awareness and sustainability prioritization (p < 0.01). Overall, the findings in Table 4 indicate that although environmental sustainability is increasingly recognized as an important component of housing development, implementation gaps remain in areas such as renewable energy integration, recycled material use, and climate-responsive construction practices.

### Comparison of sustainability perceptions between experts and the general public

An independent-samples t-test was conducted to examine differences in sustainability perceptions between expert respondents (academics and construction professionals) and the general public. The analysis revealed statistically significant differences across selected social, economic, and environmental sustainability indicators, indicating that stakeholder groups prioritize different aspects of housing sustainability within the Affordable Housing Program (AHP).

For social sustainability indicators, expert respondents assigned significantly higher importance to technical and infrastructure-related factors such as effective property management (p = 0.007), design type (p = 0.013), unit size (p < 0.001), major and minor access roads (p = 0.011), and the number of bathrooms (p = 0.016) (see Table 5). Experts also placed greater emphasis on minimizing social segregation (p = 0.024). In contrast, members of the general public assigned greater importance to community cohesion (p = 0.037) and daycare centre availability (p = 0.091), reflecting stronger concern for neighbourhood livability and family-oriented residential environments. These findings suggest that while experts focus more on planning efficiency and housing functionality, the general public prioritizes social interaction and everyday residential convenience.

**Table 5:** Comparison of experts and General public.

Designation	Experts (academia/contractors)	General public	Total	p-value
**Social Factors**				
Minimized social segregation	3.69	3.25	3.49	0.024
Lift	3.1	2.77	2.94	0.088
Design type	4.19	3.77	4	0.013
Effective property mngnt	4.66	4.35	4.52	0.007
Unit size	4.56	4.17	4.38	0.000
No. bathrooms	3.75	3.35	3.57	0.016
Daycare centre	2.94	3.29	3.1	0.091
Major/minor access road	4.45	4.09	4.29	0.011
Community cohesion	3.77	4.09	3.92	0.037
**Economic Factors**				
Cost of construction	4.59	4.34	4.48	0.083
Satisfaction of demand	4.5	3.97	4.25	0.001
**Environmental Factors**				
Noise polution	4.69	4.45	4.58	0.013
Water polution	4.77	4.56	4.67	0.008
Land use & biodiversity	4.34	4.07	4.21	0.079
Urban density	4.22	3.81	4.03	0.012
Water permeability	4.54	4.29	4.43	0.030
Waste mangnt during operation	4.75	4.53	4.64	0.017
Fresh water consumption	4.8	4.49	4.66	0.006

Economic sustainability comparisons showed fewer statistically significant differences between the two groups. However, experts expressed significantly greater concern about housing demand satisfaction (p = 0.001) and construction costs (p = 0.083), reflecting their closer engagement with housing supply systems, market constraints, and development feasibility. The general public, although also concerned with affordability, appeared more focused on practical housing accessibility and residential outcomes than broader market dynamics.

For environmental sustainability indicators, experts consistently reported higher ratings for technical environmental considerations, including noise pollution control (p = 0.013), water pollution management (p = 0.008), urban density (p = 0.012), water permeability (p = 0.030), waste management during operation (p = 0.017), and fresh water consumption (p = 0.006). Significant differences were also observed for land use and biodiversity considerations (p = 0.079). These results indicate that respondents with technical and professional backgrounds demonstrate stronger awareness of environmental planning and resource management issues. In contrast, the general public tends to prioritize environmental factors with immediate implications for residential comfort and livability.

### Factor analysis and generalized linear model

To examine the underlying structure of sustainability indicators, the study employed Principal Component Analysis (PCA) as a data reduction technique. PCA was selected to identify latent dimensions within the social, economic, and environmental sustainability variables and to reduce redundancy among correlated indicators. Before extraction, the suitability of the dataset for factor analysis was assessed using the Kaiser–Meyer–Olkin (KMO) measure of sampling adequacy and Bartlett’s Test of Sphericity. The overall KMO value of 0.812 exceeded the recommended threshold of 0.70, while Bartlett’s Test was statistically significant (p < 0.001), confirming that the correlation matrix was appropriate for component extraction. Components with eigenvalues greater than 1.0 were retained, and varimax rotation was applied to improve the interpretability of the extracted components. The PCA results identified seven social sustainability components, explaining 63.27% of the total variance; four economic sustainability components, explaining 60.50% of the variance; and six environmental sustainability components, explaining 65.88% of the variance. The extracted components represented meaningful sustainability dimensions: accessibility, infrastructure and services, affordability, housing quality, environmental performance, and community well-being.

The social sustainability PCA results indicated that accessibility to services, neighbourhood safety, housing quality, and community amenities were the dominant dimensions influencing respondents’ perceptions of sustainable housing. Economic sustainability components were primarily associated with housing affordability, construction and maintenance costs, income stability, employment opportunities, and mortgage accessibility. Environmental sustainability components addressed waste management, indoor environmental quality, water conservation, energy efficiency, sustainable material use, and pollution control. Collectively, these findings demonstrate that respondents’ sustainability perceptions were multidimensional, extending beyond physical housing attributes to encompass broader social, economic, and environmental considerations. The extracted factor scores from the PCA were subsequently used as dependent variables in the generalized linear models (GLMs) presented in Tables 6–8.

**Table 6:** GLM model for social factors.

Variable	S1 (Accessibility)	S2 (Services)	S3 (Safety)	S4 (Housing Quality)	S5 (Community)	S6 (Reputation)	S7 (Amenities)
(Intercept)	−0.593	−0.720*	0.390	−0.909*	−0.080	0.709*	0.027
factor(Education)Certificate	0.510	0.710*	−0.281	0.187	0.381	−0.202	−0.150
factor(Education)Diploma	0.581	0.534	−0.435	0.307	0.357	−0.388	−0.125
factor(Education)Bachelors	0.523	0.425	−0.494	0.478	0.195	−0.579	−0.082
factor(Education)Masters	0.612	0.277	−0.531	0.852*	0.204	−0.405	−0.223
factor(Education)Post-Graduate	0.770*	0.327	−0.366	−0.034	0.799*	−1.027*	0.389
factor(Education)PhD	−0.193	0.797	−0.412	0.863*	0.545	0.050	−0.290
factor(Designation)Contractor	0.111	−0.013	−0.454*	0.416*	−0.134	−0.270	0.134
factor(Designation)Public	−0.139	−0.070	−0.617***	0.143	0.174	−0.163	0.281
factor(Gender)Female	0.088	0.220	−0.170	0.007	−0.051	−0.107	0.025
factor(Age)31–40	0.014	0.158	0.501*	0.603**	−0.024	−0.136	−0.192
factor(Age)41–50	0.171	0.342	0.800**	0.114	−0.445*	0.017	−0.151
factor(Age)51–60	0.554*	1.036***	0.856**	0.284	−0.491	−0.260	−0.519*
factor(Age)>60	1.567**	1.176*	0.189	0.480	−1.003*	0.361	−0.398
factor(Years)10–15 years	−0.005	0.000	−0.201	0.139	−0.243	−0.013	0.052
factor(Years)16–20 years	0.044	−0.238	0.026	0.386	0.253	0.186	−0.089
factor(Years)21–25 years	−0.341	−0.803**	−0.081	−0.184	0.124	−0.118	0.429
factor(Years)26–30 years	−0.395	−0.399	0.187	0.540	0.332	−0.630	0.652
factor(Years)> 30 years	−1.922***	0.0181	−0.505	−0.189	0.308	−0.754	0.945*

Note: Coefficients represent regression estimates from generalized linear models. Reference categories are omitted to avoid multicollinearity. Statistical significance levels are indicated using asterisks: *p* < 0.05 (*), p < 0.01 (), p < 0.001 (*). S1–S7 represent extracted social sustainability factors from principal component analysis (PCA**).**

**Table 7:** GLM model for economic factors.

Parameters	ECO1	ECO2	ECO3	ECO4
(Intercept)	−1.033*	−0.925*	−0.352	−0.620
factor(Education)Certificate	0.355	1.053**	0.411	−0.032
factor(Education)Diploma	0.436	0.863*	0.465	0.379
factor(Education)Bachelors	0.525	0.660*	0.461	0.408
factor(Education)Masters	0.723.	1.018**	0.495	0.169
factor(Education)Post-Graduate	0.519	−0.277	0.441	0.640
factor(Education)PhD	1.164*	0.905*	0.248	0.588
factor(Designation)Contractor	0.487*	0.092	0.102	0.237
factor(Designation)Public	0.477**	−0.246	−0.230	0.040
factor(Gender)Female	0.032	0.032	−0.128	0.252*
factor(Age)31–40	0.522*	0.339*	0.104	0.200
factor(Age)41–50	0.080	0.418*	−0.001	0.651*
factor(Age)51–60	−0.129	0.395	0.161	0.985**
factor(Age)>60	−0.511	1.586**	1.016*	1.499**
factor(Years)10–15 years	−0.138	−0.034	0.001	−0.401*
factor(Years)16–20 years	0.440	0.228	−0.147	−0.468
factor(Years)21–25 years	0.160	−0.275	0.101	−0.769**
factor(Years)26–30 years	0.783.	−0.305	−0.178	−0.906*
factor(Years)> 30 years	0.116	−0.923*	−0.778	−1.070*

**Note:** Coefficients represent regression estimates from generalized linear models. Reference categories are omitted to avoid multicollinearity. Statistical significance levels are indicated using asterisks: *p* < 0.05 (*), p < 0.01 (****), p < 0.001 (***). ECO1–ECO4 represent extracted economic sustainability factors from principal component analysis (PCA).

**Table 8:** GLM model for Environmental factors.

	ENV1	ENV2	ENV3	ENV4	ENV5	ENV6
(Intercept)	0.119	−0.288	−0.578	−0.603	−0.572	−0.549
factor(Education)Certificate	0.292	0.368	0.520	0.669	−0.298	0.440
factor(Education)Diploma	0.586	0.321	0.554	0.417	0.271	0.590
factor(Education)Bachelors	0.359	0.323	0.482	0.472	0.554	0.576
factor(Education)Masters	0.335	0.471	0.351	0.369	0.293	0.701*
factor(Education)Post-Graduate	0.466	0.181	0.154	0.824*	0.611	0.117
factor(Education)PhD	1.289*	0.800	0.462	1.019*	1.040*	−0.004
factor(Designation)Contractor	−0.112	0.283	0.201	0.320	0.240	−0.127
factor(Designation)Public	0.011	−0.149	0.188	0.195	0.050	−0.414*
factor(Gender)Female	−0.241*	−0.014	−0.038	−0.069	0.236	0.177
factor(Age)31–40	−0.361*	−0.171	−0.303	−0.040	0.164	0.096
factor(Age)41–50	−0.742**	0.101	−0.079	−0.327	−0.176	−0.176
factor(Age)51–60	−1.075***	0.210	0.105	−0.455	0.133	0.170
factor(Age)>60	−1.183*	0.786	0.738	−0.954*	−0.113	0.593
factor(Years)10–15 years	−0.195	0.012	0.262	0.171	0.206	0.341
factor(Years)16–20 years	0.368	−0.073	0.358	0.375	0.104	0.433
factor(Years)21–25 years	0.571*	−0.119	0.016	0.681*	−0.206	0.163
factor(Years)26–30 years	0.645	−0.757*	0.270	0.806*	−0.045	0.363
factor(Years)> 30 years	0.773	−0.328	−0.987*	0.848	0.502	−0.289

**Note:** Coefficients represent regression estimates from generalized linear models. Reference categories are omitted to avoid multicollinearity. Statistical significance levels are indicated using asterisks: *p* < 0.05 (*), p < 0.01 (****), p < 0.001 (***). ENV1–ENV6 represent extracted environmental sustainability factors from principal component analysis (PCA).

Generalized Linear Models (GLMs) were fitted to assess the influence of socio-demographic characteristics on the extracted sustainability dimensions. The PCA-derived factor scores served as continuous dependent variables, while education level, professional designation, gender, age category, and years of experience were included as categorical predictor variables. Dummy coding was applied for all categorical variables, and reference categories were specified as follows: no formal education for education level, private sector for professional designation, male for gender, 18–30 years for age category, and 0–9 years for years of experience. The reported regression coefficients therefore, represent the effect of each category relative to its corresponding reference group. Statistical significance was evaluated at p < 0.05, p < 0.01, and p < 0.001 levels.

The GLM results presented in Tables 6–8 indicate that education level was one of the strongest predictors of sustainability perceptions across all three domains. Respondents with postgraduate and PhD qualifications prioritized housing quality, environmental performance, and affordability-related dimensions significantly more than those with lower educational attainment. Age also significantly influenced sustainability perceptions, particularly in the social and environmental dimensions, where older respondents placed greater emphasis on accessibility, public services, and safety-related factors. Professional designations influenced several economic and social dimensions, with contractors emphasizing cost efficiency and public officers placing greater emphasis on infrastructure and regulatory considerations. Gender differences were comparatively smaller, although female respondents showed stronger concern for selected environmental sustainability indicators. Overall, the findings suggest that perceptions of housing sustainability are shaped not only by structural housing conditions but also by respondents’ socio-demographic and professional characteristics.

## Discussion

This study aimed to assess the sustainability of Kenya’s Affordable Housing Program (AHP) in Meru and Embu counties, focusing on its economic, social, and environmental dimensions. The findings highlight both progress and persistent structural challenges that must be addressed for AHP to achieve long-term sustainability aligned with SDG 11 and Kenya’s Vision 2030 agenda.

### Social sustainability performance

The results show that social sustainability is the most developed dimension of the AHP in both counties. Safety and security were consistently rated highest among social indicators, consistent with prior research highlighting safety as a central determinant of residential satisfaction in urban housing systems [43]. Access to utilities such as electricity, water, and health facilities was also highly valued, reinforcing the importance of integrated infrastructure provision in housing delivery.

These patterns reflect broader structural inequalities in urban service provision typical of rapidly urbanizing contexts. According to [22], uneven urban infrastructure development remains a key constraint in achieving inclusive housing outcomes in developing economies, where housing delivery often outpaces service provision.

### County-level differences: Urban development and planning context

The differences between Embu and Meru can be explained by their distinct urbanization trajectories and land-use governance structures. Embu County exhibits a more consolidated urban development pattern, influenced by stronger integration into regional transport networks and relatively more structured land use planning enforcement. This has contributed to improved infrastructure density and more stable service provision systems. As a result, residents place a higher value on environmental quality, neighbourhood aesthetics, and spatial livability.

In contrast, Meru County reflects a more transitional urbanization pathway characterized by dispersed peri-urban expansion, uneven infrastructure distribution, and slower alignment between housing development and service provision. Outlook [6] highlights that such transitional urban systems often prioritize basic service delivery needs such as water access, road infrastructure, and electricity reliability over environmental amenities. This explains why Meru respondents emphasized functional infrastructure more strongly than aesthetic or environmental attributes.

These findings align with [43], who argue that differences in planning systems and urban maturity significantly shape resilience priorities and housing expectations in developing urban contexts.

### Demographic influences and behavioural drivers

Demographic characteristics significantly shaped sustainability preferences, reflecting deeper socio-economic and behavioural mechanisms documented in the literature. Recent studies underscore the importance of demographic factors in shaping sustainability perceptions, as individual preferences are influenced by differences in access to knowledge, social roles, and lived environmental risks [16,17].

Age differences reflect life-cycle housing priorities. Older respondents prioritized safety, stability, and long-term housing security, which can be explained by increased vulnerability to environmental and infrastructural risks as well as a stronger preference for reliability over innovation. In contrast, younger respondents showed a greater inclination toward smart housing features and shared amenities, reflecting increased exposure to digital technologies and evolving urban lifestyles [44].

Gender differences were particularly pronounced. Women placed greater emphasis on safety, sanitation, lighting, and access to water. Gendered roles and vulnerabilities in urban environments can explain this. Women are more likely to bear responsibility for household management and unpaid care work, which increases their dependence on reliable water, sanitation, and energy services [45,46]. In addition, women face higher exposure to safety risks in poorly serviced or inadequately lit environments, which explains their stronger prioritization of security-related housing features [47]. These structural inequalities make women more sensitive to deficiencies in basic infrastructure and neighbourhood safety.

Education emerged as a key determinant of preferences for environmental sustainability. Respondents with higher levels of education were significantly more likely to prioritize energy efficiency, waste management, and environmentally sustainable materials. This can be attributed to increased environmental awareness, greater access to information, and a better understanding of the long-term sustainability benefits. Empirical studies show that education enhances environmental competence across cognitive, behavioural, and attitudinal dimensions, leading to stronger pro-environmental decision-making [16,18]. Furthermore, more educated individuals are more likely to consider lifecycle costs and environmental externalities, rather than focusing solely on immediate affordability.

These findings demonstrate that sustainability preferences are not uniform but are shaped by socially embedded roles, access to knowledge, and differential exposure to environmental and economic risks.

### Economic sustainability and structural constraints

Affordability was the dominant economic concern across both counties, yet satisfaction levels remained low, highlighting a persistent affordability gap in Kenya’s housing delivery system [2]. This challenge reflects broader macroeconomic and structural constraints. The African Development Bank [8] notes that housing affordability in African economies is heavily influenced by rising construction costs, reliance on imported materials, and constrained housing finance systems. Similarly, [4] highlights that financialization and financing structures in housing development often limit access for low-income households.

Differences across stakeholder groups further reinforce this interpretation. Public officials emphasized policy alignment, while contractors highlighted material costs and supply chain instability. This reflects a well-documented policy–implementation gap in infrastructure delivery systems in developing economies.

### Environmental sustainability and the implementation gap

Despite strong policy-level support for sustainability principles [1], environmental integration remains the AHP’s weakest dimension. Respondents valued waste management and air quality but expressed dissatisfaction with renewable energy adoption and the use of local or green building materials. This gap reflects both institutional and structural constraints rather than the absence of materials or technologies.

Kibert [21] emphasizes that sustainable construction depends not only on material availability but also on contractor awareness, enforcement mechanisms, and lifecycle cost considerations. Similarly, [48] argues that decarbonization in developing countries is constrained by weak regulatory enforcement and limited green supply chains. Thus, low uptake of sustainable materials reflects systemic inefficiencies rather than purely technical limitations.

### Integrated demographic effects and policy implications

A key contribution of this study is the demonstration that sustainability preferences are multidimensional and socially conditioned. Generalized Linear Model (GLM) results confirm that education is the strongest predictor of environmental and economic prioritization, while gender and age significantly shape social sustainability preferences.

These findings align with [22], which emphasizes that urban housing outcomes are increasingly shaped by demographic heterogeneity rather than uniform income categories. Similarly, [17] argues that sustainable development transitions require differentiated policy instruments rather than one-size-fits-all approaches.

### County-based policy implications

Finally, the differences between Embu and Meru highlight the importance of spatially differentiated housing policy design. Meru’s emphasis on affordability and infrastructure reflects its semi-rural and transitional urban condition, while Embu’s focus on aesthetics and environmental quality reflects a more consolidated urban environment.

These findings support [43,44], who argue that urban planning systems must be context-sensitive and adapted to local governance capacity, spatial structure, and development stage. Decentralized housing governance, therefore, becomes essential for improving responsiveness, sustainability, and equity in housing delivery systems.

## Conclusion

This study evaluated the sustainability of Kenya’s Affordable Housing Program (AHP) in Meru and Embu counties by examining economic, social, and environmental sustainability dimensions using descriptive statistics, Principal Component Analysis (PCA), and Generalized Linear Models (GLMs). The findings indicate that the program has achieved moderate progress in improving social sustainability outcomes, particularly in relation to safety, electricity access, healthcare accessibility, and general service provision. However, important challenges remain in achieving balanced sustainability across all dimensions. Economic sustainability concerns were strongly associated with housing affordability, construction costs, household income levels, and limited access to mortgage financing. In contrast, environmental sustainability indicators revealed relatively low integration of renewable energy systems, recycled materials, and other green building practices. These findings suggest that although the AHP has improved housing access, significant gaps persist in affordability and environmental performance.

The study further demonstrates that sustainability perceptions vary significantly across demographic and professional groups. Education level, age, gender, and professional role influenced how respondents prioritized housing sustainability indicators, while notable differences between Embu and Meru counties highlighted the importance of local development contexts and infrastructure conditions. Older respondents placed greater emphasis on safety and accessibility, whereas more educated respondents showed stronger concern for environmental sustainability and long-term housing performance. These findings underscore the importance of adopting context-sensitive and demographic-responsive housing strategies rather than relying on uniform national approaches to affordable housing delivery.

The findings have important implications for housing policy and planning in Kenya. First, stronger integration of environmental sustainability measures is needed through enforceable green building standards, incentives for renewable energy adoption, and increased use of sustainable construction materials. Second, affordability should be addressed more comprehensively by considering not only initial housing prices but also mortgage accessibility, maintenance costs, utility expenses, and long-term financial sustainability for low- and middle-income households. The findings also highlight the importance of institutionalizing Post Occupancy Evaluation (POE) frameworks to support continuous monitoring of housing performance, resident satisfaction, and infrastructure adequacy. In addition, greater stakeholder and community participation in housing design, location selection, and infrastructure planning would improve housing uptake and long-term satisfaction among beneficiaries.

Beyond its practical implications, this study contributes to the growing literature on sustainable housing by providing one of the few empirically grounded sub-national assessments of the Affordable Housing Program in Kenya. Unlike many previous studies that rely on aggregated national data, this research demonstrates how regional context and socio-demographic characteristics shape sustainability perceptions and housing priorities. Methodologically, the study also illustrates the value of integrating PCA and GLMs in housing sustainability research to identify latent sustainability dimensions and evaluate demographic influences on housing preferences and perceptions.

Despite these contributions, the study has several limitations. The use of purposive sampling limits the generalizability of findings beyond the selected counties, although it enabled the inclusion of respondents with relevant experience and knowledge of the housing sector. In addition, the cross-sectional nature of the study limited the ability to assess changes in sustainability outcomes over time. Future research should therefore adopt longitudinal approaches to evaluate the long-term performance of Affordable Housing Program projects, particularly in relation to occupancy patterns, infrastructure provision, affordability outcomes, and environmental performance. Expanding future studies to additional counties and incorporating qualitative methods such as interviews and focus group discussions would also provide deeper insights into residents’ lived experiences and housing satisfaction.
