# Supplementary material for: Sustainability of affordable housing programs in Kenya: A case of Meru and Embu Counties
Source: PLoS One. 2026 Jul 16;21(7):e0343915. doi: 10.1371/journal.pone.0343915 (PMC13375021; doi:10.1371/journal.pone.0343915)
Supplement: Attachment — Submitted filename: Response to Reviewers.pdf [file pone.0343915.s002.pdf]

Response to Reviewers

Manuscript Title: Sustainability of Affordable Housing Programs in Kenya: A Case of Meru and Embu Counties
Manuscript ID: PONE-D-26-06731

Dear Dr. Rajan Parajuli and Reviewers,

We sincerely thank the Academic Editor and the three reviewers for their thoughtful, constructive, and detailed comments on our manuscript. We greatly appreciate the time and expertise invested in evaluating our work. The feedback has been invaluable in helping us strengthen the clarity, methodological rigor, and overall quality of the manuscript.

We have carefully addressed each comment raised by the Editor and reviewers. Below, we provide a point-by-point response. All revisions are highlighted in the marked-up version of the manuscript, and page/line numbers refer to the revised manuscript with tracked changes.

Responses to Journal Requirements

Journal Requirement 1

Comment: Please ensure that your manuscript meets PLOS ONE's style requirements, including those for file naming.

Response: We have carefully reviewed and formatted the manuscript according to PLOS ONE's style requirements, including section organization, table formatting, referencing style, and file naming conventions. The revised manuscript adheres to the specified templates.

Journal Requirement 2

Comment: Please provide additional details regarding participant consent. In the ethics statement in the Methods and online submission information, please ensure that you have specified (1) whether consent was informed and (2) what type you obtained (for instance, written or verbal, and if verbal, how it was documented and witnessed). If your study included minors, state whether you obtained consent from parents or guardians. If the need for consent was waived by the ethics committee, please include this information.

Response: We thank the reviewer for this important clarification. The manuscript has been revised to provide comprehensive details regarding participant consent. Specifically:

- Informed consent was obtained from all participants prior to data collection.
- Written consent was obtained from respondents who completed face-to-face questionnaires.
- Electronic informed consent was obtained from respondents who participated via Google Forms, with a mandatory consent acknowledgment section at the beginning of the online questionnaire.
- All participants were adults aged 18 years and above; no minors were included in the study.
- Participation was entirely voluntary, and respondents were assured of confidentiality and anonymity throughout the study.

These revisions appear in the **Data Collection Procedure** section of the Methods.

Journal Requirement 3

Comment: Thank you for stating the following financial disclosure: "University of Embu internal grant". Please state what role the funders took in the study. If the funders had no role, please state: "The funders had no role in study design, data collection and analysis, decision to publish, or preparation of the manuscript."

Response: This work was supported by the University of Embu Internal Grant (Award Number: VC-001), awarded to Karambu Kiende Gatimbu. The funders had no role in study design, data collection and analysis, decision to publish, or preparation of the manuscript.

Journal Requirement 4

Comment: When completing the data availability statement of the submission form, you indicated that you will make your data available on acceptance. We strongly recommend all authors decide on a data sharing plan before acceptance. Please note that, though access restrictions are acceptable now, your entire data will need to be made freely accessible if your manuscript is accepted for publication.

Response: The dataset underlying the findings of this study has been deposited in Zenodo and is publicly accessible through the following DOI: https://doi.org/10.5281/zenodo.18515136.

Journal Requirement 5

Comment: If the reviewer comments include a recommendation to cite specific previously published works, please review and evaluate these publications to determine whether they are relevant and should be cited.

Response: We have carefully reviewed all citation recommendations from the reviewers and have incorporated relevant and appropriate citations throughout the revised manuscript. Citations that were not directly relevant to the associated statements have been removed or replaced.

Responses to the Academic Editor

Editor Comment: Three reviewers provided very insightful comments and suggestions. I agree with them wholly. While Reviewer 3 is quite critical, I believe authors could address these comments if you carefully work on them, specifically fixing statistical analyses and the overall structure of the paper.

Response: We sincerely thank the Editor for this constructive guidance. In response, we have undertaken substantial revisions throughout the manuscript:

1. **Statistical analyses:** We identified and corrected data entry errors in two economic sustainability variables where values had been mistakenly entered as "55" instead of "5", causing implausible mean values exceeding the 1–5 Likert scale. All analyses (descriptive statistics, PCA, GLMs, t-tests, ANOVA) have been rerun with the corrected dataset. The revised results now fall within valid ranges, and all tables have been updated accordingly. Statistical procedures have been described with greater rigor and transparency.

2. **Manuscript structure:** The Introduction, Literature Review, and Conceptual Framework sections have been restructured to improve logical flow and reduce repetition. The conceptual framework has been refined to clearly explain how the three sustainability dimensions informed the study design and analytical approach.

3. **Language quality:** The manuscript has undergone thorough language editing to improve grammar, coherence, and scientific presentation.

We believe these revisions have substantially strengthened the scientific rigor and clarity of the manuscript.

Responses to Reviewer #1

Reviewer #1 General Comment: Overall, this is a timely study that tackles a key pillar of the SDGs and Kenya's Vision 2030.

Response: We sincerely thank the reviewer for recognizing the relevance and timeliness of our study.

Comment 1: The authors have talked of "limited use of local or green materials" without providing details whether these are available and if so, why are they not being used.

Response: We appreciate this important observation. The Discussion section has been expanded to explain that while Kenya possesses locally available sustainable building materials—including stabilized soil blocks, bamboo, pozzolanic cement substitutes, recycled construction aggregates, and agricultural waste-based materials—their adoption in the Affordable Housing Program remains limited. We now discuss several contributing factors: (1) weak enforcement of existing sustainability standards and building codes; (2) higher upfront cost perceptions despite long-term lifecycle savings; (3) limited technical awareness and training among local contractors; (4) market preferences for conventional materials (e.g., cement, steel) driven by familiarity and perceived reliability; and (5) supply chain constraints for alternative materials. Supporting literature has been added to substantiate these explanations.

Location in revised manuscript: Discussion section, paragraphs 5-6.

Comment 2: The authors should enrich the discussion section by providing socio-economic-environmental rationales with relevant citations in literature that support such arguments to explain the observed results and the differences between the two counties of Embu and Meru. It is only through such lens that this study would be valuable for policy makers going forward to make improvements that address the shortcomings of the AHP or concerns arising from this study and the research community, in this area of work. For instance, why did the women give higher importance to safety, sanitation, and waste management? What is driving such inclination in these counties and what do we find in the scientific literature in this matter?

Response: We thank the reviewer for this valuable suggestion. The Discussion section has been substantially expanded to interpret the findings within the socio-economic and environmental contexts of Embu and Meru counties. Specifically:

County-level differences: We now explain that Embu County exhibits a more consolidated urban development pattern due to stronger integration into regional transport networks and more structured land use planning enforcement, which has contributed to improved infrastructure density and stable service provision. In contrast, Meru County reflects a more transitional urbanization pathway characterized by dispersed peri-urban expansion and uneven infrastructure distribution. These structural differences explain why Meru respondents emphasized basic service accessibility while Embu respondents prioritized environmental quality and neighborhood aesthetics. Citations from Asadzadeh et al. (2022) and Nesmachnow & Callejo (2025) have been added.

Gender-based differences: We now explain that women's stronger prioritization of safety, sanitation, lighting, and water access is linked to gendered roles and vulnerabilities in urban environments. Women bear disproportionate responsibility for household management and unpaid care work (Tacoli, 2012; Bradshaw et al., 2019), which increases their dependence on reliable water, sanitation, and energy services. Additionally, women face higher exposure to safety risks in poorly serviced or inadequately lit environments (UN Women, 2021), explaining their stronger prioritization of security-related housing features. These structural inequalities make women more sensitive to deficiencies in basic infrastructure.

Education-based differences: We explain that respondents with postgraduate education showed stronger preference for environmental sustainability due to increased environmental awareness, access to information, and greater understanding of long-term sustainability benefits. Education enhances environmental competence across cognitive, behavioral, and attitudinal dimensions (Borgonovi et al., 2022; Sahu et al., 2024), leading to stronger pro-environmental decision-making.

Location in revised manuscript: Discussion section, subsections "County-level differences: urban development and planning context" and "Demographic influences and behavioural drivers."

Comment 3: Pg 2 Ln 29-31 and pg 4 ln 109-110 are almost similar. Consider revising these to avoid duplication.

Response: We thank the reviewer for identifying this repetition. The duplicated statements have been revised and consolidated. The abstract has been streamlined, and the redundant sentence in the Introduction has been removed or rephrased to eliminate repetition while preserving necessary information.

Location in revised manuscript: Abstract (page 2) and Introduction (page 4).

Comment 4: Pg 4 ln 118: the authors talk of lack of enforcement of green building codes. Do we have green building codes in Kenya? If so, the authors should highlight this policy provision, if not, then this is irrelevant here.

Response: We appreciate this clarification. The manuscript has been revised to accurately reflect Kenya's policy landscape. Kenya has sustainability-related policy provisions and building guidelines that promote green construction practices, including the Kenya Building Code reforms (under development), environmental regulations under the National Environment Management Authority (NEMA), and the Kenya Green Building Society (KGBS) rating tools. However, a fully comprehensive and legally enforceable national green building code does not yet exist. We have revised the discussion to avoid implying the existence of a complete national code while acknowledging existing policy frameworks that support sustainable construction. The relevant sentence has been rephrased to state: "weak enforcement of existing sustainability standards and building guidelines."

Location in revised manuscript: Discussion section, paragraph 5.

Comment 5: Pg 7 Ln 189-194: This research involves human subjects that requires Institutional Review Board (IRB) approval for its validity to be published in an international journal. The IRB number and where it can be obtained should be provided here despite the statements on pg 7 ln 208-214 (Data Collection Procedure).

Response: We thank the reviewer for raising this important ethical consideration. We acknowledge that formal IRB approval is standard practice for human subjects' research. We provide the following clarification:

This study involved non-sensitive survey data focusing on perceptions of housing sustainability and did not involve medical, clinical, or highly sensitive personal information. At the time of the study (data collection conducted February–March 2021), the institutional ethics framework at the University of Embu was still under development, and formal IRB/NACOSTI approval was not sought because the research was considered low risk and observational in nature.

Nevertheless, strict ethical standards were observed throughout the study. Informed consent was obtained from all participants prior to data collection, participation was voluntary, anonymity was guaranteed, and no personally identifiable information was collected. These ethical safeguards have been clearly described in the revised manuscript.

We recognize the importance of formal ethics approval for future research and have noted this as a limitation in the revised manuscript. We have also added a statement that the study adhered to the principles of the Declaration of Helsinki.

Location in revised manuscript: Data Collection Procedure section and Limitations subsection of Conclusion.

Comment 6: Pg 7 Ln 216: Provide citation for Cronbach's alpha.

Response: We have added appropriate methodological citations for Cronbach's alpha reliability analysis. The following references have been included: Ahmad et al. (2024) and Izah et al. (2023).

Location in revised manuscript: Reliability and Validity of the Instrument subsection.

Comment 7: Pg 7 Ln 221: Provide citation for exploratory factor analysis (EFA).

Response: We have added relevant citations supporting the use of Exploratory Factor Analysis, including Hair et al. (2019) and Field (2024).

Location in revised manuscript: Data Analysis Techniques subsection.

Comment 8: Pg 7 Ln 224-234 (Data Analysis Techniques): This is a critical part of any research, yet it lacks details that would enable replication of the analysis by someone else other than the co-authors. Please provide concise details on these techniques (EFA, PCA, GLMs, thematic analysis, etc.) with appropriate citations and why they were the most suited for this work. Provide which version of Stata was used, etc. Such details are standard in methods section.

Response: We thank the reviewer for this important methodological critique. The Data Analysis Techniques section has been substantially revised to improve transparency and reproducibility. We now provide:

- **STATA version:** STATA 18 was used for all quantitative analyses.
- **EFA justification and specification:** We explain that EFA was used to identify latent constructs underlying participant perceptions, with the model equation provided, KMO and Bartlett's test results, and varimax rotation procedures.
- **PCA justification and specification:** We clarify that PCA was used for data reduction to construct composite indices, with Kaiser's eigenvalue >1 criterion.
- **GLM justification and specification:** We explain that GLMs were chosen due to their flexibility in handling non-normal response distributions and Likert-scale

data as approximately continuous variables, with the model equation and link function specified.

- **t-test and ANOVA justification:** We explain that independent samples t-tests compare means between two groups, while one-way ANOVA compares means across multiple groups, with post-hoc Tukey HSD tests where applicable.
- **Thematic analysis:** We describe the inductive-deductive coding approach using NVivo 12, following Braun & Clarke's (2006) framework.

All techniques are now accompanied by appropriate methodological citations and clear justifications for their selection.

Location in revised manuscript: Data Analysis Techniques subsection (substantially expanded).

Comment 9: Pg 9 Table 2: It is not clear what column "sig" stands for, but my guess is it is p-value. Same to Tables 3-4.

Response: The column label "sig" has been replaced with "p-value" in all relevant tables (Tables 2, 3, and 4) for clarity.

Location in revised manuscript: Tables 2, 3, and 4.

Comment 10: Pg 9 Ln 252: I cannot see the asterisks in Table 2, which I believe is what is being referenced here.

Response: We apologize for this formatting issue. The table formatting has been revised, and the asterisks indicating statistical significance levels (* $p < 0.05$, ** $p < 0.01$, *** $p < 0.001$) are now clearly visible in all tables. The reference in the text has been corrected accordingly.

Location in revised manuscript: Table 2 and accompanying text.

Comment 11: Pg 13 Table 5: I cannot see factor(Education)secondary here; my guess is that since "education" was dummy variable, one had to be dropped to avoid dummy variable trap, yet I am unable to see where this is discussed. Additionally, Table 5 is not well labelled. What is column 1 and what are S1-S7? Tables should be self-contained with sufficient information. What do the asterisk mean? Same comments to Tables 6-7.

Response: We thank the reviewer for this important clarification. The following revisions have been made:

1. **Reference category clarification:** We have explicitly stated in the text that "no formal education" was used as the reference category for education level, "private sector" for professional designation, "male" for gender, "18-30 years" for age category, and "0-9 years" for years of experience. This explains why "secondary" education does not appear as a separate coefficient (it is included in the reference category).
2. **Table labeling:** Tables 5-7 have been substantially revised to be self-contained. Table headings now clearly indicate that S1-S7 represent extracted social sustainability factors from PCA, ECO1-ECO4 represent economic sustainability factors, and ENV1-ENV6 represent environmental sustainability factors. A note has been added to each table explaining the abbreviations and the meaning of asterisks.
3. **Asterisks explanation:** A legend has been added to each table: "* p < 0.05, ** p < 0.01, *** p < 0.001".

Location in revised manuscript: GLM section (preceding Tables 5-7) and Tables 5, 6, and 7.

Comment 12: Pg 15 Ln 319-322: Discussion is a critical part of any research as this is where the results and interpreted in the context of the results. Authors should provide further details on the underlying differences between the two counties that explain the results differences. For instance, what are the "counties' distinct stages of urban development and land use planning" between Embu and Meru?

Response: We thank the reviewer for this excellent suggestion. The Discussion section now includes a detailed subsection titled "County-level differences: urban development and planning context" that explains:

- Embu County's more consolidated urban development pattern, influenced by stronger integration into regional transport networks (especially proximity to the Nairobi-Meru highway corridor) and more structured land use planning enforcement. This has resulted in improved infrastructure density, more stable service provision systems, and greater emphasis on environmental quality and neighborhood aesthetics.
- Meru County's more transitional urbanization pathway, characterized by dispersed peri-urban expansion, uneven infrastructure distribution, and slower alignment between housing development and service provision. This explains why Meru respondents emphasized basic service accessibility (water, electricity, transport connectivity) over environmental amenities.

These explanations are supported by recent literature (Asadzadeh et al., 2022; Outlook, 2022).

Location in revised manuscript: Discussion section, "County-level differences: urban development and planning context" subsection.

Comment 13: Pg 16 Ln 358-362: Highlight and cite the recent studies that underscore the importance of demographics in shaping sustainability preferences. See my general comments on what needs to be added. There is so much underlying factors that explain these results, which should be discussed here. What are informing such patterns?

Response: We have substantially expanded this part of the Discussion. Recent studies examining demographic influences on sustainability perceptions and housing preferences have been added (Borgonovi et al., 2022; Sahu et al., 2024; Bizikova, 2023). We now provide detailed explanations of the underlying factors informing observed patterns:

- **Age differences:** Older respondents prioritize safety and infrastructure reliability due to increased vulnerability to environmental and infrastructural risks and stronger preference for stability over innovation. Younger respondents show greater inclination toward smart housing features due to increased digital technology exposure.
- **Gender differences:** Women's stronger prioritization of safety, sanitation, lighting, and water access reflects gendered household responsibilities and higher exposure to safety risks in poorly serviced environments.
- **Education differences:** Higher education enhances environmental awareness, access to sustainability information, and consideration of lifecycle costs and environmental externalities.

Location in revised manuscript: Discussion section, "Demographic influences and behavioural drivers" subsection.

Responses to Reviewer #2

Comment 1: Introduction: Sustainability in this context is not limited to the physical durability of structures but includes social inclusivity, economic viability, and environmental stewardship. Where is the source of this information? The paragraph on, despite its ambitious goals, the AHP faces complex challenges needs some editing. You have stated only one challenge; how about other challenges and implications of these

challenges? Lines 40 and 41 require citations. The paragraph on lines 42 to 52 also requires relevant citations.

Response: We thank the reviewer for these constructive suggestions. The Introduction has been substantially revised:

1. **Sustainability definition source:** We have added citations to the widely recognized three-pillar framework of sustainable development from the World Commission on Environment and Development (1987) and Purvis et al. (2019).
2. **AHP challenges expansion:** The paragraph on AHP challenges has been expanded to include multiple interconnected challenges: affordability concerns (housing costs exceeding financial capacity), inadequate financing mechanisms and limited access to affordable mortgage products, land acquisition constraints, bureaucratic delays, inconsistent provision of essential services (water, sanitation, electricity), poor environmental integration, and inadequate community participation. Citations have been added for each challenge.
3. **Citation additions:** Relevant citations have been added throughout the Introduction to support all factual claims, including housing deficit statistics, urbanization projections, and sustainability frameworks.

Location in revised manuscript: Introduction, paragraphs 2-4.

Comment 2: Research gaps and emerging issues: You have not cited the studies for instance: this statement "Limited research explores the influence of participatory planning and community engagement on project sustainability. Most studies focus on economic sustainability alone, neglecting the interconnectedness of social and environmental dimensions." After this sentence, the authors could have included the citations. Even in the next sentence, "Despite the growing body of research, several gaps persist..." Please put citations after the sentences.

Response: We apologize for this oversight. Appropriate citations have been added throughout the Research Gaps and Emerging Issues section to support all claims regarding existing knowledge gaps. Citations now follow each gap statement.

Location in revised manuscript: Research Gaps and Emerging Issues subsection.

Comment 3: Conceptual framework: You have only highlighted what entails economic, social, and environmental sustainability. How about the importance of those variables, and what about the indicators used in the results? Where is the source of the indicators and their importance? You can check the article on the sustainability assessment of

residential neighbourhood with varying densities in Nairobi, Kenya and see how they had organized the indicators. You also highlight that you are using sustainability indicators.

Response: We thank the reviewer for this valuable guidance. The Conceptual Framework section has been substantially revised to:

1. Explain the importance of each sustainability dimension and how they contribute to overall housing sustainability outcomes.
2. Clearly list the indicators used for each dimension, with sources from established housing sustainability literature.
3. Reference the recommended article (Dadzie et al., 2018) as well as other key sources (Kibert, 2016; UN-Habitat, 2020) that informed indicator selection.
4. Explain how the conceptual framework guided data collection and analysis.

The revised section now provides a clear, synthesized framework that directly links the theoretical dimensions to the empirical indicators presented in the results.

Location in revised manuscript: Conceptual Framework subsection.

Comment 4: Data analysis techniques: Justify the use of independent samples t-tests and ANOVA.

Response: We have revised the Data Analysis Techniques section to include explicit justifications for the use of independent samples t-tests and one-way ANOVA. Specifically:

- **Independent samples t-test:** Used to compare mean sustainability perception scores between two independent groups (e.g., Meru vs. Embu counties; experts vs. general public). This test is appropriate because the dependent variables (sustainability indices) are continuous and approximately normally distributed.
- **One-way ANOVA:** Used to compare means across more than two independent groups (e.g., age categories, education levels, professional roles). ANOVA controls for Type I error inflation that would arise from multiple pairwise t-tests. Post-hoc Tukey HSD tests are applied following significant ANOVA results.

Location in revised manuscript: Data Analysis Techniques subsection.

Comment 5: Target population: The manuscript indicates that the sample included both experts and members of the general public; however, the results presented appear to

focus mainly on expert responses. The results from the general public (e.g., community representatives) are not clearly presented or discussed. In my opinion, the perceptions of the general public should be analyzed and presented separately from those of experts, as these two groups are likely to have different priorities. The authors are therefore encouraged to clearly present the results of the general public, compare them with expert results, and discuss any differences between the two groups.

Response: We thank the reviewer for this excellent and important recommendation. In response, we conducted independent samples t-tests comparing expert respondents (academics and construction professionals, n=115) with members of the general public (n=100). The revised manuscript now includes:

1. **A new table** presenting the comparison of sustainability perceptions between experts and the general public across social, economic, and environmental indicators.
2. **Detailed results** showing statistically significant differences between the two groups.
3. **Discussion** of the observed differences and their implications for housing policy.

Key findings include:

- Experts placed significantly higher importance on technical factors: effective property management ($p=0.007$), design type ($p=0.013$), unit size ($p<0.001$), construction costs ($p=0.083$), and environmental management indicators (noise pollution $p=0.013$, water pollution $p=0.008$, urban density $p=0.012$).
- The general public prioritized community cohesion ($p=0.037$) and daycare facilities ($p=0.091$), reflecting stronger concern for neighborhood livability and family-oriented environments.
- Both groups shared strong concern regarding affordability and basic service accessibility.

These additions significantly strengthen the inclusiveness and analytical depth of the study.

Location in revised manuscript: Results section (new table and text), Discussion section.

Comment 6: Results: Kindly explain what S1, S2, and S3 mean in Table 5, same as in Tables 6 and 7.

Response: Tables 5-7 have been revised to be self-contained. Table headings now clearly indicate that:

- **S1-S7** represent extracted social sustainability factors from Principal Component Analysis (e.g., S1 = Accessibility, S2 = Services, S3 = Safety, S4 = Housing Quality, S5 = Community, S6 = Reputation, S7 = Amenities).
- **ECO1-ECO4** represent extracted economic sustainability factors.
- **ENV1-ENV6** represent extracted environmental sustainability factors.

A note has been added to each table explaining these abbreviations.

Location in revised manuscript: Tables 5, 6, and 7.

Comment 7: Discussion: Recent studies underscore the importance of demographics in shaping sustainability preferences (citations for this). The present study showed that respondents with postgraduate education were significantly more likely to value environmental features such as energy efficiency and waste separation. Why? Women prioritized household safety, access to lighting, and proximity to water sources, while men placed more weight on affordability and ownership potential. Why?

Response: The Discussion section has been expanded to explain these demographic differences using recent literature and contextual interpretation. A new subsection "Demographic influences and behavioural drivers" now provides:

- **Citations** for recent studies on demographics and sustainability preferences (Borgonovi et al., 2022; Sahu et al., 2024; Bizikova, 2023).
- **Explanation for education differences:** Respondents with postgraduate education show stronger environmental preferences due to increased environmental awareness, access to information, and greater understanding of long-term sustainability benefits. Education enhances environmental competence across cognitive, behavioral, and attitudinal dimensions, and more educated individuals are more likely to consider lifecycle costs and environmental externalities rather than focusing solely on immediate affordability.
- **Explanation for gender differences:** Women's prioritization of safety, sanitation, lighting, and water access reflects gendered household responsibilities (Tacoli, 2012; Bradshaw et al., 2019). Women bear disproportionate responsibility for household management and unpaid care work, increasing their dependence on reliable water, sanitation, and energy services. Additionally, women face higher exposure to safety risks in poorly serviced or inadequately lit environments (UN Women, 2021).

Location in revised manuscript: Discussion section, "Demographic influences and behavioural drivers" subsection.

Comment 8: Conclusion: Put some recommendations.

Response: The Conclusion section has been revised to include practical policy recommendations, including:

- Stronger integration of environmental sustainability measures through enforceable green building standards and incentives for renewable energy adoption.
- Comprehensive affordability strategies addressing mortgage accessibility, maintenance costs, utility expenses, and long-term financial sustainability.
- Institutionalization of Post Occupancy Evaluation (POE) frameworks.
- Greater stakeholder and community participation in housing design, location selection, and infrastructure planning.
- Decentralization of housing planning and implementation to enable local adaptation.

Location in revised manuscript: Conclusion section, paragraphs 3-4.

Responses to Reviewer #3

Comment 1: Introduction: Some statements regarding Kenya's housing challenges are presented without supporting citations. Please provide relevant literature or official sources to substantiate these claims. The final sentence of the Introduction states: "Subsequent parts of the paper are structured as follows: 'Materials and Methods' section describes ..." However, the section that follows is "Literature Review," not "Materials and Methods." Please revise this part for consistency with the actual structure of the manuscript.

Response: We thank the reviewer for these careful observations.

- **Citations added:** Additional citations from peer-reviewed literature, government reports (Kenya National Bureau of Statistics, 2022), and housing policy documents have been incorporated throughout the Introduction to substantiate all claims regarding Kenya's housing challenges.
- **Manuscript structure corrected:** The final sentence of the Introduction has been revised to accurately reflect the manuscript's organization: "The remainder

> of the paper is structured as follows: the *Literature Review* section examines existing studies on housing sustainability and policy frameworks; the *Materials and Methods* section describes the study design, data collection, and analytical techniques; the *Results and Discussion* section presents and interprets the findings; and the *Conclusion and Recommendations* section provides the final synthesis and policy implications."

Location in revised manuscript: Introduction, final paragraph.

Comment 2: Literature Review - Reference [6]: This section opens with the statement "Sustainable housing theory is embedded within the broader concept". This appears abrupt and conceptually unclear. In addition, the cited reference [6], Creswell, J. W., & Creswell, J. D. (2018). Research design, is a general research design textbook and does not appear to support this statement.

Response: We agree with the reviewer's critique. The opening statement has been revised for conceptual clarity. More importantly, the inappropriate citation to Creswell & Creswell has been removed and replaced with the correct citation to the World Commission on Environment and Development (1987), which introduced the foundational definition of sustainable development. The revised sentence now reads: "Sustainable housing is grounded in the broader concept of sustainable development, commonly defined as development that meets present needs without compromising the ability of future generations to meet their own needs (World Commission on Environment and Development, 1987)."

Location in revised manuscript: Literature Review, Theoretical Framework subsection.

Comment 3: Literature Review - Reference [7]: The statement "the triadic model of sustainability—economic, social, and environmental dimensions—which together provide a holistic understanding of long-term housing viability" is supported by reference [7]. Please check whether this citation is appropriate and revise either the statement or the supporting reference accordingly.

Response: We thank the reviewer for this careful checking. The citation has been corrected. The triadic model of sustainability is now appropriately cited to Purvis et al. (2019) and Sachs (2015), which discuss the three pillars of sustainable development. The reference to Kibert (2016) has been retained for sustainable construction applications of the framework.

Location in revised manuscript: Literature Review, Theoretical Framework subsection.

Comment 4: Literature Review - Reference [8, 9]: [8] Etikan, I., Musa, S. A., & Alkassim, R. S. (2016). Comparison of convenience sampling and purposive sampling. [9] Fox, J. (2015). Applied regression analysis and generalized linear models. These two papers are cited in relation to the conceptual framework of sustainable housing, but both sources appear to concern research methodology rather than housing theory or sustainability concepts. These citations do not seem appropriate in this context. More broadly, the manuscript contains several citations whose connection to the associated statements is unclear. This raises concerns about the accuracy of citation use and, consequently, about the overall reliability of the manuscript.

Response: We thank the reviewer for this rigorous and important critique. We have conducted a comprehensive review of all citations throughout the manuscript to ensure each reference appropriately supports its associated statement. Specifically:

- The citations to Etikan et al. (2016) and Fox (2015) have been removed from the Literature Review and Conceptual Framework sections, as they were indeed inappropriate in those contexts.
- Etikan et al. (2016) has been relocated to the Sampling Technique subsection of the Methods, where it appropriately supports the discussion of purposive sampling.
- Fox (2015) has been relocated to the Data Analysis Techniques subsection, where it appropriately supports the discussion of Generalized Linear Models.

We have carefully cross-checked every citation in the revised manuscript to ensure alignment with the associated arguments. A citation map was created to verify that each reference is used in an appropriate context. We believe this has substantially improved the scholarly rigor of the manuscript.

Location in revised manuscript: Sampling Technique subsection; Data Analysis Techniques subsection.

Comment 5: Conceptual Framework: This section includes substantial background and contextual explanation that would be better placed in the Introduction. In its current form, the section reads more like a selective listing of loosely connected studies than a clearly synthesized conceptual framework. The authors are encouraged to reorganize this part and clarify how the cited literature informs the present study.

Response: We thank the reviewer for this constructive critique. The Conceptual Framework section has been completely restructured to:

1. **Provide a clear synthesis** rather than a selective listing of studies.
2. **Explicitly state** how the three sustainability dimensions (economic, social, environmental) inform the study's research questions, data collection instruments, and analytical approach.
3. **Explain the mediating role** of demographic characteristics in shaping sustainability perceptions.
4. **Present a clear conceptual model** showing the relationships between sustainability dimensions, demographic variables, and sustainability outcomes.

Background and contextual explanations that were better suited for the Introduction have been moved. The revised section now reads as a focused, synthesized framework that directly guides the empirical investigation.

Location in revised manuscript: Conceptual Framework subsection (substantially restructured).

Comment 6: Overall structure and writing style: The manuscript has substantial problems in basic scholarly writing and presentation. The text is often repetitive, stylistically uneven, and at times grammatically awkward. Several sections read as descriptive compilations rather than as a focused scientific argument. The manuscript requires major language editing and careful restructuring before its scientific contribution can be properly evaluated.

Response: We acknowledge the reviewer's concerns regarding language quality and presentation. The manuscript has undergone substantial language editing and restructuring. Specifically:

- **Professional language editing:** The manuscript was reviewed by a native English speaker with expertise in academic writing. Grammar, syntax, and style have been improved throughout.
- **Redundancy elimination:** Repetitive passages have been consolidated or removed. For example, the abstract and introduction previously contained overlapping statements; these have been revised for distinctiveness.
- **Structural improvements:** Sections have been reorganized to improve logical flow. The Introduction now moves from broad context (urbanization, housing deficit) to specific program (AHP) to study objectives. The Literature Review

now moves from theoretical foundation to conceptual framework to research gaps.

- **Focused scientific argument:** Each section now clearly advances the central argument. Descriptive compilation passages have been replaced with analytical synthesis.

We believe the revised manuscript now meets the scholarly writing standards expected for publication.

Comment 7: Statistical and methodological concerns: The survey design and statistical analysis also require substantial clarification. The study uses purposive sampling with a heterogeneous group of respondents, yet the conclusions are sometimes presented too broadly relative to what this design can support. In addition, the measurement framework is insufficiently described, and some reported results appear internally inconsistent, most notably the presence of mean values exceeding the stated 1–5 Likert scale.

Response: We thank the reviewer for identifying these critical issues. We have addressed each concern as follows:

1. Purposive sampling and generalizability: We have revised the Conclusion to explicitly acknowledge the limitations of purposive sampling and the associated constraints on generalizability beyond the selected counties. We now state: "The use of purposive sampling limits the generalizability of findings beyond the selected counties, although it enabled the inclusion of respondents with relevant experience and knowledge of the housing sector." We have also been careful throughout the manuscript to avoid overgeneralizing conclusions beyond what the study design supports.

2. Mean values exceeding Likert scale: Upon re-examination of the dataset, we identified data entry errors in two economic sustainability variables ("Housing affordability" and "Cost of construction"), where some responses had mistakenly been entered as "55" instead of "5". The dataset has been corrected, and all analyses (descriptive statistics, PCA, GLMs, t-tests, ANOVA) have been rerun with the corrected data. The revised results now fall within the valid 1–5 Likert scale range. Corresponding tables (especially Table 3) have been updated, and interpretations have been revised accordingly.

3. Measurement framework: The measurement framework has been substantially expanded in the "Instrumentation and Data Collection Tools" subsection, including detailed descriptions of the five questionnaire sections, Likert-scale items, and the pilot testing procedure.

Location in revised manuscript: Instrumentation subsection; Data Analysis Techniques subsection; Conclusion (limitations).

Comment 8: The statistical methods are also not reported with sufficient rigor. The manuscript does not clearly distinguish between exploratory factor analysis and principal component analysis, and the factor analytic results are presented without enough detail to assess their validity. Likewise, the GLM analysis is insufficiently specified, making it difficult to interpret the reported coefficients and to evaluate the reliability of the findings. Overall, the methodological and statistical sections require major revision before the results can be properly assessed.

Response: We thank the reviewer for this critical methodological assessment. The Data Analysis Techniques section has been substantially revised to provide rigorous and transparent reporting:

1. Distinction between EFA and PCA: We now clearly explain that:

- **EFA** was used to identify latent constructs underlying participant perceptions of sustainability indicators, with the model equation provided and rotation methods specified.
- **PCA** was subsequently applied as a data reduction technique to simplify the dataset and support construction of composite indices. We clarify that PCA is a variable reduction technique while EFA is a latent construct identification technique.

2. Factor analytic results detail: We now provide:

- KMO measure of sampling adequacy (overall 0.812) and Bartlett's Test of Sphericity results ($p < 0.001$).
- Eigenvalues and variance explained for each extracted component (social: 7 components explaining 63.27%; economic: 4 components explaining 60.50%; environmental: 6 components explaining 65.88%).
- Description of varimax rotation and factor loading interpretation.

3. GLM specification: We now provide:

- The full GLM equation with link function specified.
- Clarification that a Gaussian family with identity link was used for continuous outcomes.
- Dummy coding description for all categorical variables.

- Reference categories explicitly stated.
- Interpretation guidance for regression coefficients.

4. Software version: STATA 18 is explicitly stated.

We believe these revisions provide sufficient detail for replication and assessment of statistical validity.

Location in revised manuscript: Data Analysis Techniques subsection (substantially expanded).

We are deeply grateful to the Academic Editor and all three reviewers for their thorough, constructive, and insightful comments. The revision process has substantially improved the clarity, methodological rigor, theoretical grounding, and policy relevance of our manuscript. We believe the revised manuscript now meets the publication standards of PLOS ONE.

We respectfully submit the revised manuscript and accompanying materials for your consideration.

Sincerely,

Dr. Karambu Kiende Gatimbu (Corresponding Author)
Department of Business Studies, University of Embu, Embu, Kenya
Email: kiende.gatimbu@embuni.ac.ke

On behalf of all co-authors
