# Supplementary material for: Sustainability of affordable housing programs in Kenya: A case of Meru and Embu Counties
Source: PLoS One. 2026 Jul 16;21(7):e0343915. doi: 10.1371/journal.pone.0343915 (PMC13375021; doi:10.1371/journal.pone.0343915)
Supplement: Peer review history

# Peer review: Decision Letter 0

**DOI:** 10.1371/journal.pone.0343915.r001

**Authors:** Rajan Parajuli

8 Apr 2026

PONE-D-26-06731

Sustainability of Affordable Housing Programs in Kenya: A case of Meru and Embu Counties

PLOS One

Dear Dr. Gatimbu,

Thank you for submitting your manuscript to PLOS ONE. After careful consideration, we feel that it has merit but does not fully meet PLOS ONE’s publication criteria as it currently stands. Therefore, we invite you to submit a revised version of the manuscript that addresses the points raised during the review process.

Please submit your revised manuscript by May 23 2026 11:59PM. If you will need more time than this to complete your revisions, please reply to this message or contact the journal office at plosone@plos.org. When you're ready to submit your revision, log on to https://www.editorialmanager.com/pone/ and select the ’submissions Needing Revision' folder to locate your manuscript file.

Please include the following items when submitting your revised manuscript:

- A letter that responds to each point raised by the academic editor and reviewer(s). You should upload this letter as a separate file labeled 'Response to Reviewers'.
- A marked-up copy of your manuscript that highlights changes made to the original version. You should upload this as a separate file labeled 'Revised Manuscript with Track Changes'.
- An unmarked version of your revised paper without tracked changes. You should upload this as a separate file labeled 'Manuscript'.

If you would like to make changes to your financial disclosure, please include your updated statement in your cover letter. Guidelines for resubmitting your figure files are available below the reviewer comments at the end of this letter.

If applicable, we recommend that you deposit your laboratory protocols in protocols.io to enhance the reproducibility of your results. Protocols.io assigns your protocol its own identifier (DOI) so that it can be cited independently in the future. For instructions see: https://journals.plos.org/plosone/s/submission-guidelines#loc-laboratory-protocols. Additionally, PLOS ONE offers an option for publishing peer-reviewed Lab Protocol articles, which describe protocols hosted on protocols.io. Read more information on sharing protocols at https://plos.org/protocols?utm_medium=editorial-email&utm_source=authorletters&utm_campaign=protocols.

As the corresponding author, your ORCID iD is verified in the submission system and will appear in the published article. PLOS supports the use of ORCID, and we encourage all coauthors to register for an ORCID iD and use it as well. Please encourage your coauthors to verify their ORCID iD within the submission system before final acceptance, as unverified ORCID iDs will not appear in the published article. *Only*  the individual author can complete the verification step; PLOS staff *cannot*  verify ORCID iDs on behalf of authors.

We look forward to receiving your revised manuscript.

Kind regards,

Rajan Parajuli

Academic Editor

PLOS One

Journal Requirements:

When submitting your revision, we need you to address these additional requirements.

1. Please ensure that your manuscript meets PLOS ONE’s style requirements, including those for file naming. The PLOS ONE style templates can be found at

https://journals.plos.org/plosone/s/file?id=wjVg/PLOSOne_formatting_sample_main_body.pdf and

https://journals.plos.org/plosone/s/file?id=ba62/PLOSOne_formatting_sample_title_authors_affiliations.pdf

2. Please provide additional details regarding participant consent. In the ethics statement in the Methods and online submission information, please ensure that you have specified (1) whether consent was informed and (2) what type you obtained (for instance, written or verbal, and if verbal, how it was documented and witnessed). If your study included minors, state whether you obtained consent from parents or guardians. If the need for consent was waived by the ethics committee, please include this information.

If you are reporting a retrospective study of medical records or archived samples, please ensure that you have discussed whether all data were fully anonymized before you accessed them and/or whether the IRB or ethics committee waived the requirement for informed consent. If patients provided informed written consent to have data from their medical records used in research, please include this information.

3. Thank you for stating the following financial disclosure:

"University of Embu internal grant"

Please state what role the funders took in the study.  If the funders had no role, please state: "The funders had no role in study design, data collection and analysis, decision to publish, or preparation of the manuscript."

If this statement is not correct you must amend it as needed.

Please include this amended Role of Funder statement in your cover letter; we will change the online submission form on your behalf.

4. When completing the data availability statement of the submission form, you indicated that you will make your data available on acceptance. We strongly recommend all authors decide on a data sharing plan before acceptance, as the process can be lengthy and hold up publication timelines. Please note that, though access restrictions are acceptable now, your entire data will need to be made freely accessible if your manuscript is accepted for publication. This policy applies to all data except where public deposition would breach compliance with the protocol approved by your research ethics board. If you are unable to adhere to our open data policy, please kindly revise your statement to explain your reasoning and we will seek the editor’s input on an exemption. Please be assured that, once you have provided your new statement, the assessment of your exemption will not hold up the peer review process.

5. If the reviewer comments include a recommendation to cite specific previously published works, please review and evaluate these publications to determine whether they are relevant and should be cited. There is no requirement to cite these works unless the editor has indicated otherwise.

Additional Editor Comments:

Three reviewers provided very insightful comments and suggestions. I agree with them wholly. While Reviewer 3 is quite critical, I believe authors could address these comments if you carefully work on them, specifically fixing statistical analyses and the overall structure of the paper.

[Note: HTML markup is below. Please do not edit.]

Reviewers' comments:

Reviewer’s Responses to Questions

**Comments to the Author**

1. Is the manuscript technically sound, and do the data support the conclusions?

The manuscript must describe a technically sound piece of scientific research with data that supports the conclusions. Experiments must have been conducted rigorously, with appropriate controls, replication, and sample sizes. The conclusions must be drawn appropriately based on the data presented.

Reviewer #1: Yes

Reviewer #2: Yes

Reviewer #3: No

**********

2. Has the statistical analysis been performed appropriately and rigorously?

Reviewer #1: Yes

Reviewer #2: Yes

Reviewer #3: No

**********

3. Have the authors made all data underlying the findings in their manuscript fully available?

The PLOS Data policy requires authors to make all data underlying the findings described in their manuscript fully available without restriction, with rare exception (please refer to the Data Availability Statement in the manuscript PDF file). The data should be provided as part of the manuscript or its supporting information, or deposited to a public repository. For example, in addition to summary statistics, the data points behind means, medians and variance measures should be available. If there are restrictions on publicly sharing data—e.g. participant privacy or use of data from a third party—those must be specified.

Reviewer #1: No

Reviewer #2: Yes

Reviewer #3: Yes

**********

4. Is the manuscript presented in an intelligible fashion and written in standard English?

PLOS ONE does not copyedit accepted manuscripts, so the language in submitted articles must be clear, correct, and unambiguous. Any typographical or grammatical errors should be corrected at revision, so please note any specific errors here.

Reviewer #1: Yes

Reviewer #2: No

Reviewer #3: No

**********

5. Review Comments to the Author

Please use the space provided to explain your answers to the questions above. You may also include additional comments for the author, including concerns about dual publication, research ethics, or publication ethics. (Please upload your review as an attachment if it exceeds 20,000 characters)

Reviewer #1: PONE-D-26-06731

General comments:

Overall, this is a timely study that tackles a key pillar of the SDGs and Kenya’s Vision 2030.c

The authors have talked of “limited use of local or green materials” without proving details whether these are available and if so, why are they not being used.

The authors should enrich the discussion section by proving social-economic-environmental rationales with relevant citations in literature that support such arguments to explain the observed results and the differences between the two counties of Embu and Meru. It is only through such lens that this study would be valuable for policy makers going forward to make improvements that address the shortcomings of the AHP or concerns arising from this study and the research community, in this area of work. For instance, why did the women give higher importance to safety, sanitation, and waste management? What is driving such inclination in these counties and what do we find in the scientific literature in this matter?

Specific Comments:

Pg 2 Ln 29-31 and pg 4 ln 109-110 are almost similar. Consider revising these to avoid duplication.

Pg 4 ln 118: the authors talk of lack of enforcement of green building codes. Do we have green building codes in Kenya? If so, the authors should highlight this policy provision, if not, then this is irrelevant here.

Pg 7 Ln 189-194: This research involves human subjects that requires Institutional Review Board (IRB) approval for its validity to be published in an international journal. The IRB number and where it can be obtained should be provided here despite the statements on pg 7 ln 208-214 (Data Collection Procedure).

Pg 7 Ln 216: Provide citation for Cronbach’s alpha.

Pg 7 Ln 221: Provide citation for exploratory factor analysis (EFA).

Pg 7 Ln 224-234 (Data Analysis Techniques): This is a critical part of any research, yet it lacks details that would enable replication of the analysis by someone else other than the co-authors. Please provide concise details on these techniques (EFA, PCA, GLMs, thematic analysis, etc.) with appropriate citations and why they were the most suited for this work. Provide which version of Stata was used, etc. Such details are standard in methods section.

Pg 9 Table 2: It is not clear what column “sig” stands for, but my guess is it is p-value. Same to Tables 3-4

Pg 9 Ln 252: I cannot see the asterisks in Table 2, which I believe is what is being referenced here.

Pg 13 Table 5: I cannot see factor(Education)secondary here; my guess is that since “education” was dummy variable, one had to be dropped to avoid dummy variable trap, yet I am unable to see where this is discussed. Additionally, Table 5 is not well labelled. What is column 1 and what are S1-S7? Tables should be self-contained with sufficient information. What do the asterisk mean? Same comments to Tables 6-7.

Pg 15 Ln 319-322: Discussion is a critical part of any research as this is where the results and interpreted in the context of the results. Authors should provide further details on the underlying differences between the two counties that explain the results differences. For instance, what are the “counties’ distinct stages of urban development and land use planning” between Embu and Meru?

Pg 16 Ln 358-362: Highlight and cite the recent studies that underscore the importance of demographics in shaping sustainability preferences. See my general comments on what needs to be added. There is so much underlying factors that explain these results, which should be discussed here. What are informing such patterns?

Reviewer #2: Thank you for submitting your research. Please find below my comments for each section.

Introduction

Sustainability in this context is not limited to the physical durability of structures but includes social inclusivity, economic viability, and environmental stewardship. Where is the source of this information?

The paragraph on, despite its ambitious goals, the AHP faces complex challenges needs some editing. You have stated only one challenge; how about other challenges and implications of these challenges?

Lines 40 and 41 require citations. The paragraph on lines 42 to 52 also requires relevant citations.

Research gaps and emerging issues

You have not cited the studies for instance: this statement Limited research explores the influence of participatory planning and community engagement on project sustainability. Most studies focus on economic sustainability alone, neglecting the interconnectedness of social and environmental dimensions. After this sentence, the authors could have included the citations. Even in the next sentence, “Despite the growing body of research, several gaps persist. First, most studies focus on Nairobi and Mombasa, neglecting semi-urban counties like Meru and Embu. Second, there is limited exploration of how demographic variables influence perceptions of sustainability. Third, existing evaluations rarely triangulate between economic, social, and environmental dimensions, often treating them in isolation. Moreover, while sustainability policies exist on paper, few studies have measured their implementation effectiveness at the county level. There is also minimal attention paid to the role of professional stakeholders such as contractors and academics in shaping housing outcomes. Please put citations after the sentences.

Conceptual framework

You have only highlighted what entails economic, social, and environmental sustainability. How about the importance of those variables, and what about the indicators used in the results? Where is the source of the indicators and their importance? You can check the article on the sustainability assessment of residential neighbourhoods with varying densities in Nairobi, Kenya and see how they had organized the indicators. You also highlight that you are using sustainability indicators.

Data analysis techniques

Justify the use of independent samples t-tests and ANOVA

Target population

The manuscript indicates that the sample included both experts and members of the general public; however, the results presented appear to focus mainly on expert responses. The results from the general public (e.g., community representatives) are not clearly presented or discussed. In my opinion, the perceptions of the general public should be analyzed and presented separately from those of experts, as these two groups are likely to have different priorities. The general public are the potential beneficiaries of the AHP housing evaluation and may place more importance on factors such as affordability, accessibility to services, and neighborhood livability, while experts may focus more on technical and planning criteria.

The authors are therefore encouraged to clearly present the results of the general public, compare them with expert results, and discuss any differences between the two groups. You can check how the authors framed the opinions from the residents in this article, "Comparative Analysis of Household Residential Preferences Across Neighbourhood Densities in Nairobi, Kenya."

Results

Kindly explain what S1, S2, and S3 mean in Table 5, same as in Tables 6 and 7

Discussion

Recent studies underscore the importance of demographics in shaping sustainability preferences (citations for this). The present study showed that respondents with postgraduate education were significantly more likely to value environmental features such as energy efficiency and waste separation. Why?

Women prioritized household safety, access to lighting, and proximity to water sources, while men placed more weight on affordability and ownership potential. Why?

Conclusion

Put some recommendations.

Reviewer #3: 1. Introduction:

Some statements regarding Kenya’s housing challenges are presented without supporting citations. Please provide relevant literature or official sources to substantiate these claims.

The final sentence of the Introduction states: “Subsequent parts of the paper are structured as follows: ‘Materials and Methods’ section describes ...” However, the section that follows is “Literature Review,” not “Materials and Methods.” Please revise this part for consistency with the actual structure of the manuscript.

2. Literature Review

- Reference [6]

This section opens with the statement "Sustainable housing theory is embedded within the broader concept". This appears abrupt and conceptually unclear. In addition, the cited reference [6], Creswell, J. W., & Creswell, J. D. (2018). Research design, is a general research design textbook and does not apper to support this statement.

- Reference [7]

The statement "the triadic model of sustainability economic, social, and environmental dimensions which together provide a holistic understanding of long-term housing viability" is supported by reference [7]. lease check whether this citation is appropriate and revise either the statement or the supporting reference accordingly.

- Reference [8, 9]

[8] Etikan, I., Musa, S. A., & Alkassim, R. S. (2016). Comparison of convenience sampling and purposive sampling. American Journal of Theoretical and Applied Statistics, 5(1), 1–4.

[9] Fox, J. (2015). Applied regression analysis and generalized linear models (3rd ed.). Sage Publications.

These two papers are cited in relation to the conceptual framework of sustainable housing, but both sources appear to concern research methodology rather than housing theory or sustainability concepts. These citations do not seem appropriate in this context. More broadly, the manuscript contains several citations whose connection to the associated statements is unclear. This raises concerns about the accuracy of citation use and, consequently, about the overall reliability of the manuscript.

3. Conceptual Framework

This section includes substantial background and contextual explanation that would be better placed in the Introduction. In its current form, the section reads more like a selective listing of loosely connected studies than a clearly synthesized conceptual framework. The authors are encouraged to reorganize this part and clarify how the cited literature informs the present study.

4. Overall structure and writing style

Pointing out similar problems in every section would be extensive and beyond the practical scope of peer review. I therefore focus here on the broader concerns.

The manuscript has substantial problems in basic scholarly writing and presentation. The text is often repetitive, stylistically uneven, and at times grammatically awkward. Several sections read as descriptive compilations rather than as a focused scientific argument. In addition, there are inconsistencies between the stated methods and the reported analyses, and at least some numerical results appear implausible (for example, mean values exceeding the stated 1–5 Likert scale). The manuscript requires major language editing and careful restructuring before its scientific contribution can be properly evaluated.

5. Statistical and methodological concerns

The survey design and statistical analysis also require substantial clarification. The study uses purposive sampling with a heterogeneous group of respondents, yet the conclusions are sometimes presented too broadly relative to what this design can support. In addition, the measurement framework is insufficiently described, and some reported results appear internally inconsistent, most notably the presence of mean values exceeding the stated 1–5 Likert scale.

The statistical methods are also not reported with sufficient rigor. The manuscript does not clearly distinguish between exploratory factor analysis and principal component analysis, and the factor analytic results are presented without enough detail to assess their validity. Likewise, the GLM analysis is insufficiently specified, making it difficult to interpret the reported coefficients and to evaluate the reliability of the findings. Overall, the methodological and statistical sections require major revision before the results can be properly assessed.

**********

6. PLOS authors have the option to publish the peer review history of their article (what does this mean?). If published, this will include your full peer review and any attached files.

If you choose “no”, your identity will remain anonymous but your review may still be made public.

**Do you want your identity to be public for this peer review?**  For information about this choice, including consent withdrawal, please see our Privacy Policy.

Reviewer #1: No

Reviewer #2: No

Reviewer #3: No

**********

[NOTE: If reviewer comments were submitted as an attachment file, they will be attached to this email and accessible via the submission site. Please log into your account, locate the manuscript record, and check for the action link "View Attachments". If this link does not appear, there are no attachment files.]

To ensure your figures meet our technical requirements, please review our figure guidelines: https://journals.plos.org/plosone/s/figures

You may also use PLOS’s free figure tool, NAAS, to help you prepare publication quality figures: https://journals.plos.org/plosone/s/figures#loc-tools-for-figure-preparation.

NAAS will assess whether your figures meet our technical requirements by comparing each figure against our figure specifications.

# Author comment: Author response to Decision Letter 1

**DOI:** 10.1371/journal.pone.0343915.r002

5 Jun 2026

Response to Reviewers

Manuscript Title: Sustainability of Affordable Housing Programs in Kenya: A Case of Meru and Embu Counties Manuscript ID: PONE-D-26-06731

Dear Dr. Rajan Parajuli and Reviewers,

We sincerely thank the Academic Editor and the three reviewers for their thoughtful, constructive, and detailed comments on our manuscript. We greatly appreciate the time and expertise invested in evaluating our work. The feedback has been invaluable in helping us strengthen the clarity, methodological rigor, and overall quality of the manuscript.

We have carefully addressed each comment raised by the Editor and reviewers. Below, we provide a point-by-point response. All revisions are highlighted in the marked-up version of the manuscript, and page/line numbers refer to the revised manuscript with tracked changes.

Responses to Journal Requirements

Journal Requirement 1

Comment: Please ensure that your manuscript meets PLOS ONE’s style requirements, including those for file naming.

Response: We have carefully reviewed and formatted the manuscript according to PLOS ONE’s style requirements, including section organization, table formatting, referencing style, and file naming conventions. The revised manuscript adheres to the specified templates.

Journal Requirement 2

Comment: Please provide additional details regarding participant consent. In the ethics statement in the Methods and online submission information, please ensure that you have specified (1) whether consent was informed and (2) what type you obtained (for instance, written or verbal, and if verbal, how it was documented and witnessed). If your study included minors, state whether you obtained consent from parents or guardians. If the need for consent was waived by the ethics committee, please include this information.

Response: We thank the reviewer for this important clarification. The manuscript has been revised to provide comprehensive details regarding participant consent. Specifically:

• Informed consent was obtained from all participants prior to data collection.

• Written consent was obtained from respondents who completed face-to-face questionnaires.

• Electronic informed consent was obtained from respondents who participated via Google Forms, with a mandatory consent acknowledgment section at the beginning of the online questionnaire.

• All participants were adults aged 18 years and above; no minors were included in the study.

• Participation was entirely voluntary, and respondents were assured of confidentiality and anonymity throughout the study.

These revisions appear in the Data Collection Procedure section of the Methods.

Journal Requirement 3

Comment: Thank you for stating the following financial disclosure: "University of Embu internal grant". Please state what role the funders took in the study. If the funders had no role, please state: "The funders had no role in study design, data collection and analysis, decision to publish, or preparation of the manuscript."

Response: This work was supported by the University of Embu Internal Grant (Award Number: VC-001), awarded to Karambu Kiende Gatimbu. The funders had no role in study design, data collection and analysis, decision to publish, or preparation of the manuscript.

Journal Requirement 4

Comment: When completing the data availability statement of the submission form, you indicated that you will make your data available on acceptance. We strongly recommend all authors decide on a data sharing plan before acceptance. Please note that, though access restrictions are acceptable now, your entire data will need to be made freely accessible if your manuscript is accepted for publication.

Response: The dataset underlying the findings of this study has been deposited in Zenodo and is publicly accessible through the following DOI: https://doi.org/10.5281/zenodo.18515136.

Journal Requirement 5

Comment: If the reviewer comments include a recommendation to cite specific previously published works, please review and evaluate these publications to determine whether they are relevant and should be cited.

Response: We have carefully reviewed all citation recommendations from the reviewers and have incorporated relevant and appropriate citations throughout the revised manuscript. Citations that were not directly relevant to the associated statements have been removed or replaced.

Responses to the Academic Editor

Editor Comment: Three reviewers provided very insightful comments and suggestions. I agree with them wholly. While Reviewer 3 is quite critical, I believe authors could address these comments if you carefully work on them, specifically fixing statistical analyses and the overall structure of the paper.

Response: We sincerely thank the Editor for this constructive guidance. In response, we have undertaken substantial revisions throughout the manuscript:

1.

Statistical analyses: We identified and corrected data entry errors in two economic sustainability variables where values had been mistakenly entered as "55" instead of "5", causing implausible mean values exceeding the 1–5 Likert scale. All analyses (descriptive statistics, PCA, GLMs, t-tests, ANOVA) have been rerun with the corrected dataset. The revised results now fall within valid ranges, and all tables have been updated accordingly. Statistical procedures have been described with greater rigor and transparency.

2.

Manuscript structure: The Introduction, Literature Review, and Conceptual Framework sections have been restructured to improve logical flow and reduce repetition. The conceptual framework has been refined to clearly explain how the three sustainability dimensions informed the study design and analytical approach.

3.

Language quality: The manuscript has undergone thorough language editing to improve grammar, coherence, and scientific presentation.

We believe these revisions have substantially strengthened the scientific rigor and clarity of the manuscript.

Responses to Reviewer #1

Reviewer #1 General Comment: Overall, this is a timely study that tackles a key pillar of the SDGs and Kenya’s Vision 2030.

Response: We sincerely thank the reviewer for recognizing the relevance and timeliness of our study.

Comment 1: The authors have talked of "limited use of local or green materials" without providing details whether these are available and if so, why are they not being used.

Response: We appreciate this important observation. The Discussion section has been expanded to explain that while Kenya possesses locally available sustainable building materials—including stabilized soil blocks, bamboo, pozzolanic cement substitutes, recycled construction aggregates, and agricultural waste-based materials—their adoption in the Affordable Housing Program remains limited. We now discuss several contributing factors: (1) weak enforcement of existing sustainability standards and building codes; (2) higher upfront cost perceptions despite long-term lifecycle savings; (3) limited technical awareness and training among local contractors; (4) market preferences for conventional materials (e.g., cement, steel) driven by familiarity and perceived reliability; and (5) supply chain constraints for alternative materials. Supporting literature has been added to substantiate these explanations.

Location in revised manuscript: Discussion section, paragraphs 5-6.

Comment 2: The authors should enrich the discussion section by providing socio-economic-environmental rationales with relevant citations in literature that support such arguments to explain the observed results and the differences between the two counties of Embu and Meru. It is only through such lens that this study would be valuable for policy makers going forward to make improvements that address the shortcomings of the AHP or concerns arising from this study and the research community, in this area of work. For instance, why did the women give higher importance to safety, sanitation, and waste management? What is driving such inclination in these counties and what do we find in the scientific literature in this matter?

Response: We thank the reviewer for this valuable suggestion. The Discussion section has been substantially expanded to interpret the findings within the socio-economic and environmental contexts of Embu and Meru counties. Specifically:

County-level differences: We now explain that Embu County exhibits a more consolidated urban development pattern due to stronger integration into regional transport networks and more structured land use planning enforcement, which has

contributed to improved infrastructure density and stable service provision. In contrast, Meru County reflects a more transitional urbanization pathway characterized by dispersed peri-urban expansion and uneven infrastructure distribution. These structural differences explain why Meru respondents emphasized basic service accessibility while Embu respondents prioritized environmental quality and neighborhood aesthetics. Citations from Asadzadeh et al. (2022) and Nesmachnow & Callejo (2025) have been added.

Gender-based differences: We now explain that women’s stronger prioritization of safety, sanitation, lighting, and water access is linked to gendered roles and vulnerabilities in urban environments. Women bear disproportionate responsibility for household management and unpaid care work (Tacoli, 2012; Bradshaw et al., 2019), which increases their dependence on reliable water, sanitation, and energy services. Additionally, women face higher exposure to safety risks in poorly serviced or inadequately lit environments (UN Women, 2021), explaining their stronger prioritization of security-related housing features. These structural inequalities make women more sensitive to deficiencies in basic infrastructure.

Education-based differences: We explain that respondents with postgraduate education showed stronger preference for environmental sustainability due to increased environmental awareness, access to information, and greater understanding of long-term sustainability benefits. Education enhances environmental competence across cognitive, behavioral, and attitudinal dimensions (Borgonovi et al., 2022; Sahu et al., 2024), leading to stronger pro-environmental decision-making.

Location in revised manuscript: Discussion section, subsections "County-level differences: urban development and planning context" and "Demographic influences and behavioural drivers."

Comment 3: Pg 2 Ln 29-31 and pg 4 ln 109-110 are almost similar. Consider revising these to avoid duplication.

Response: We thank the reviewer for identifying this repetition. The duplicated statements have been revised and consolidated. The abstract has been streamlined, and the redundant sentence in the Introduction has been removed or rephrased to eliminate repetition while preserving necessary information.

Location in revised manuscript: Abstract (page 2) and Introduction (page 4).

Comment 4: Pg 4 ln 118: the authors talk of lack of enforcement of green building codes. Do we have green building codes in Kenya? If so, the authors should highlight this policy provision, if not, then this is irrelevant here.

Response: We appreciate this clarification. The manuscript has been revised to accurately reflect Kenya’s policy landscape. Kenya has sustainability-related policy provisions and building guidelines that promote green construction practices, including the Kenya Building Code reforms (under development), environmental regulations under the National Environment Management Authority (NEMA), and the Kenya Green Building Society (KGBS) rating tools. However, a fully comprehensive and legally enforceable national green building code does not yet exist. We have revised the discussion to avoid implying the existence of a complete national code while acknowledging existing policy frameworks that support sustainable construction. The relevant sentence has been rephrased to state: "weak enforcement of existing sustainability standards and building guidelines."

Location in revised manuscript: Discussion section, paragraph 5.

Comment 5: Pg 7 Ln 189-194: This research involves human subjects that requires Institutional Review Board (IRB) approval for its validity to be published in an international journal. The IRB number and where it can be obtained should be provided here despite the statements on pg 7 ln 208-214 (Data Collection Procedure).

Response: We thank the reviewer for raising this important ethical consideration. We acknowledge that formal IRB approval is standard practice for human subjects’ research. We provide the following clarification:

This study involved non-sensitive survey data focusing on perceptions of housing sustainability and did not involve medical, clinical, or highly sensitive personal information. At the time of the study (data collection conducted February–March 2021), the institutional ethics framework at the University of Embu was still under development, and formal IRB/NACOSTI approval was not sought because the research was considered low risk and observational in nature.

Nevertheless, strict ethical standards were observed throughout the study. Informed consent was obtained from all participants prior to data collection, participation was voluntary, anonymity was guaranteed, and no personally identifiable information was collected. These ethical safeguards have been clearly described in the revised manuscript.

We recognize the importance of formal ethics approval for future research and have noted this as a limitation in the revised manuscript. We have also added a statement that the study adhered to the principles of the Declaration of Helsinki.

Location in revised manuscript: Data Collection Procedure section and Limitations subsection of Conclusion.

Comment 6: Pg 7 Ln 216: Provide citation for Cronbach’s alpha.

Response: We have added appropriate methodological citations for Cronbach’s alpha reliability analysis. The following references have been included: Ahmad et al. (2024) and Izah et al. (2023).

Location in revised manuscript: Reliability and Validity of the Instrument subsection.

Comment 7: Pg 7 Ln 221: Provide citation for exploratory factor analysis (EFA).

Response: We have added relevant citations supporting the use of Exploratory Factor Analysis, including Hair et al. (2019) and Field (2024).

Location in revised manuscript: Data Analysis Techniques subsection.

Comment 8: Pg 7 Ln 224-234 (Data Analysis Techniques): This is a critical part of any research, yet it lacks details that would enable replication of the analysis by someone else other than the co-authors. Please provide concise details on these techniques (EFA, PCA, GLMs, thematic analysis, etc.) with appropriate citations and why they were the most suited for this work. Provide which version of Stata was used, etc. Such details are standard in methods section.

Response: We thank the reviewer for this important methodological critique. The Data Analysis Techniques section has been substantially revised to improve transparency and reproducibility. We now provide:

• STATA version: STATA 18 was used for all quantitative analyses.

• EFA justification and specification: We explain that EFA was used to identify latent constructs underlying participant perceptions, with the model equation provided, KMO and Bartlett’s test results, and varimax rotation procedures.

• PCA justification and specification: We clarify that PCA was used for data reduction to construct composite indices, with Kaiser’s eigenvalue >1 criterion.

• GLM justification and specification: We explain that GLMs were chosen due to their flexibility in handling non-normal response distributions and Likert-scale

data as approximately continuous variables, with the model equation and link function specified.

• t-test and ANOVA justification: We explain that independent samples t-tests compare means between two groups, while one-way ANOVA compares means across multiple groups, with post-hoc Tukey HSD tests where applicable.

• Thematic analysis: We describe the inductive-deductive coding approach using NVivo 12, following Braun & Clarke’s (2006) framework.

All techniques are now accompanied by appropriate methodological citations and clear justifications for their selection.

Location in revised m

Attachment Submitted filename: Response to Reviewers.pdf

# Peer review: Decision Letter 1

**DOI:** 10.1371/journal.pone.0343915.r003

**Authors:** Rajan Parajuli, Rajan Parajuli

30 Jun 2026

Sustainability of Affordable Housing Programs in Kenya: A case of Meru and Embu Counties

PONE-D-26-06731R1

Dear Dr. Gatimbu,

We’re pleased to inform you that your manuscript has been judged scientifically suitable for publication and will be formally accepted for publication once it meets all outstanding technical requirements.

Within one week, you’ll receive an e-mail detailing the required amendments. When these have been addressed, you’ll receive a formal acceptance letter and your manuscript will be scheduled for publication.

An invoice will be generated when your article is formally accepted. Please note, if your institution has a publishing partnership with PLOS and your article meets the relevant criteria, all or part of your publication costs will be covered. Please make sure your user information is up-to-date by logging into Editorial Manager at Editorial Manager® and clicking the ‘Update My Information' link at the top of the page. For questions related to billing, please contact billing support.

If your institution or institutions have a press office, please notify them about your upcoming paper to help maximize its impact. If they’ll be preparing press materials, please inform our press team as soon as possible -- no later than 48 hours after receiving the formal acceptance. Your manuscript will remain under strict press embargo until 2 pm Eastern Time on the date of publication. For more information, please contact onepress@plos.org.

Kind regards,

Rajan Parajuli

Academic Editor

PLOS One

Additional Editor Comments (optional):

While reviewer 3 raised additional concerns, I believe that authors have addressed all reviewers' concerns carefully in this version.

Reviewers' comments:

Reviewer’s Responses to Questions

**Comments to the Author**

1. If the authors have adequately addressed your comments raised in a previous round of review and you feel that this manuscript is now acceptable for publication, you may indicate that here to bypass the “Comments to the Author” section, enter your conflict of interest statement in the “Confidential to Editor” section, and submit your "Accept" recommendation.

Reviewer #1: All comments have been addressed

Reviewer #2: All comments have been addressed

Reviewer #3: (No Response)

**********

2. Is the manuscript technically sound, and do the data support the conclusions?

The manuscript must describe a technically sound piece of scientific research with data that supports the conclusions. Experiments must have been conducted rigorously, with appropriate controls, replication, and sample sizes. The conclusions must be drawn appropriately based on the data presented.

Reviewer #1: Yes

Reviewer #2: Partly

Reviewer #3: No

**********

3. Has the statistical analysis been performed appropriately and rigorously?

Reviewer #1: Yes

Reviewer #2: Yes

Reviewer #3: No

**********

4. Have the authors made all data underlying the findings in their manuscript fully available?

The PLOS Data policy requires authors to make all data underlying the findings described in their manuscript fully available without restriction, with rare exception (please refer to the Data Availability Statement in the manuscript PDF file). The data should be provided as part of the manuscript or its supporting information, or deposited to a public repository. For example, in addition to summary statistics, the data points behind means, medians and variance measures should be available. If there are restrictions on publicly sharing data—e.g. participant privacy or use of data from a third party—those must be specified.

Reviewer #1: No

Reviewer #2: Yes

Reviewer #3: Yes

**********

5. Is the manuscript presented in an intelligible fashion and written in standard English?

PLOS ONE does not copyedit accepted manuscripts, so the language in submitted articles must be clear, correct, and unambiguous. Any typographical or grammatical errors should be corrected at revision, so please note any specific errors here.

Reviewer #1: Yes

Reviewer #2: Yes

Reviewer #3: Yes

**********

6. Review Comments to the Author

Please use the space provided to explain your answers to the questions above. You may also include additional comments for the author, including concerns about dual publication, research ethics, or publication ethics. (Please upload your review as an attachment if it exceeds 20,000 characters)

Reviewer #1: Thank you for addressing my comments. I am satisfied with the revised manuscript. I have no further comments.

Reviewer #2: Thank you for the opportunity to review this manuscript. Overall, the study addresses an important topic on Affordable housing and sustainability. The author has addressed all the comments from the revision

Reviewer #3: The manuscript addresses an important and policy-relevant topic, and the motivation for evaluating the sustainability of affordable housing programs in Meru and Embu counties is well justified. However, the main limitation of the study lies in the treatment of highly heterogeneous survey respondents as a single analytical sample. The manuscript appears to pool responses from residents or prospective beneficiaries, public officials, academics, planners, contractors, and other technical or institutional actors, although many survey items are likely to have different meanings depending on the respondent’s standpoint. As a result, even if the statistical procedures such as PCA, GLM, and ANOVA are technically implemented, the validity of the substantive conclusions is limited by unresolved problems of measurement equivalence, stakeholder positionality, and construct validity.

Specific concerns are as follows:

1.Stakeholder position is not sufficiently incorporated into the analysis.

The manuscript includes respondents from different groups, such as public institutions, academia, the construction sector, and community-level participants. However, it is not sufficiently clear whether respondents answered as residents, prospective beneficiaries, public officials, project implementers, contractors, planners, or researchers. These roles are not interchangeable and should be explicitly defined and incorporated into the analysis.

2. The same survey item may measure different constructs across respondent groups.

For example, safety or security may refer to lived experience and daily vulnerability for residents, but to planning standards, design features, or implementation requirements for officials or contractors. If the same numerical score reflects different meanings across groups, pooled analysis becomes difficult to interpret.

3. Economic sustainability indicators are especially standpoint-dependent.

Affordability, household income, ownership potential, construction cost, maintenance cost, and financial accessibility have different meanings for demand-side respondents and supply-side respondents. For residents or prospective beneficiaries, affordability reflects actual or anticipated ability to pay. For public officials or contractors, it may reflect whether the project meets policy targets or cost-control assumptions. These responses should not be treated as equivalent without justification.

4. Environmental sustainability indicators mix resident experience with technical assessment.

Items such as thermal comfort, noise pollution, lighting quality, and water service quality may be appropriate as resident-experience indicators. However, items such as recycled material content, cement substitutes, freshwater consumption, and energy production require technical knowledge, design documentation, construction records, environmental monitoring, or objective performance data. General survey respondents may not be competent to answer these items reliably.

5. Several environmental terms are conceptually ambiguous.

"Noise pollution" could mean noise generated by the housing project or noise exposure from the surrounding environment. "Water pollution" could refer to pollution of nearby water bodies caused by the project, or to poor household drinking-water quality. "Freshwater consumption" could refer to household water use or to water-saving design features. "Energy production" should also be clearly defined, particularly if the authors mean on-site renewable energy production. These terms need precise definitions and exact questionnaire wording.

6. The expert–general public comparison does not resolve the problem.

Table 5 compares experts and the general public, but score comparisons are meaningful only if the scores measure the same construct across groups. A difference between experts and the public may reflect different knowledge levels, different reference frames, or different interpretations of the items, rather than genuine differences in sustainability perceptions.

7. The GLM models may suffer from omitted-variable bias and construct non-equivalence.

The models include demographic variables such as age, gender, education, and income, but do not appear to adequately control for stakeholder role or direct residential experience. For example, postgraduate respondents may be disproportionately drawn from academics, public officials, or technical experts. Therefore, an apparent education effect may actually reflect stakeholder positionality or access to project information.

8. Pooling all respondents into a single model may be inappropriate.

The GLM analyses appear to assume that all respondents interpreted the dependent variables in the same way. This assumption is not justified. If affordability, comfort, safety, or environmental performance are interpreted differently by residents and project-side actors, then the estimated demographic coefficients are difficult to interpret.

9. The authors should distinguish between user-experience indicators and project-side technical indicators.

Resident-oriented items should focus on lived experience, perceived affordability, comfort, safety, service access, and satisfaction. Expert- or project-side items should focus on design standards, construction practices, material use, cost control, environmental technologies, and implementation procedures. These two types of evidence should not be pooled without conceptual justification.

**********

7. PLOS authors have the option to publish the peer review history of their article (what does this mean?). If published, this will include your full peer review and any attached files.

If you choose “no”, your identity will remain anonymous but your review may still be made public.

**Do you want your identity to be public for this peer review?**  For information about this choice, including consent withdrawal, please see our Privacy Policy.

Reviewer #1: No

Reviewer #2: No

Reviewer #3: No

**********

# Editor report: Acceptance letter

**DOI:** 10.1371/journal.pone.0343915.r004

**Authors:** Rajan Parajuli, Rajan Parajuli

PONE-D-26-06731R1

PLOS One

Dear Dr. Gatimbu,

I'm pleased to inform you that your manuscript has been deemed suitable for publication in PLOS One. Congratulations! Your manuscript is now being handed over to our production team.

At this stage, our production department will prepare your paper for publication. This includes ensuring the following:

* All references, tables, and figures are properly cited

* All relevant supporting information is included in the manuscript submission,

* There are no issues that prevent the paper from being properly typeset

You will receive further instructions from the production team, including instructions on how to review your proof when it is ready. Please keep in mind that we are working through a large volume of accepted articles, so please give us a few days to review your paper and let you know the next and final steps.

Lastly, if your institution or institutions have a press office, please let them know about your upcoming paper now to help maximize its impact. If they'll be preparing press materials, please inform our press team within the next 48 hours. Your manuscript will remain under strict press embargo until 2 pm Eastern Time on the date of publication. For more information, please contact onepress@plos.org.

You will receive an invoice from PLOS for your publication fee after your manuscript has reached the completed accept phase. If you receive an email requesting payment before acceptance or for any other service, this may be a phishing scheme. Learn how to identify phishing emails and protect your accounts at https://explore.plos.org/phishing.

If we can help with anything else, please email us at customercare@plos.org.

Thank you for submitting your work to PLOS ONE and supporting open access.

Kind regards,

PLOS ONE Editorial Office Staff

on behalf of

Dr. Rajan Parajuli

Academic Editor

PLOS One
